# Phosphoproteomic Analysis Reveals Downstream PKA Effectors of AKAP Cypher/ZASP in the Pathogenesis of Dilated Cardiomyopathy

**DOI:** 10.3389/fcvm.2021.753072

**Published:** 2021-12-13

**Authors:** Jialan Lv, Zhicheng Pan, Jian Chen, Rui Xu, Dongfei Wang, Jiaqi Huang, Yang Dong, Jing Jiang, Xiang Yin, Hongqiang Cheng, Xiaogang Guo

**Affiliations:** ^1^Department of Cardiology, The First Affiliated Hospital, Zhejiang University School of Medicine, Hangzhou, China; ^2^Department of Cardiology, The Second Affiliated Hospital, Zhejiang University School of Medicine, Hangzhou, China; ^3^Department of Physiology and Pathophysiology, Peking University Health Science Center, Beijing, China; ^4^Department of Pathology and Pathophysiology, Zhejiang University School of Medicine, Hangzhou, China

**Keywords:** Cypher/ZASP, AKAP, PKA substrates, phosphoproteomics, dilated cardiomyopathy, β-catenin

## Abstract

**Background:** Dilated cardiomyopathy (DCM) is a major cause of heart failure worldwide. The Z-line protein Cypher/Z-band alternatively spliced PDZ-motif protein (ZASP) is closely associated with DCM, both clinically and in animal models. Our earlier work revealed Cypher/ZASP as a PKA-anchoring protein (AKAP) that tethers PKA to phosphorylate target substrates. However, the downstream PKA effectors regulated by AKAP Cypher/ZASP and their relevance to DCM remain largely unknown.

**Methods and Results:** For the identification of candidate PKA substrates, global quantitative phosphoproteomics was performed on cardiac tissue from wild-type and Cypher-knockout mice with PKA activation. A total of 216 phosphopeptides were differentially expressed in the Cypher-knockout mice; 31 phosphorylation sites were selected as candidates using the PKA consensus motifs. Bioinformatic analysis indicated that differentially expressed proteins were enriched mostly in cell adhesion and mRNA processing. Furthermore, the phosphorylation of β-catenin Ser675 was verified to be facilitated by Cypher. This phosphorylation promoted the transcriptional activity of β-catenin, and also the proliferative capacity of cardiomyocytes. Immunofluorescence staining demonstrated that Cypher colocalised with β-catenin in the intercalated discs (ICD) and altered the cytoplasmic distribution of β-catenin. Moreover, the phosphorylation of two other PKA substrates, vimentin Ser72 and troponin I Ser23/24, was suppressed by Cypher deletion.

**Conclusions:** Cypher/ZASP plays an essential role in β-catenin activation *via* Ser675 phosphorylation, which modulates cardiomyocyte proliferation. Additionally, Cypher/ZASP regulates other PKA effectors, such as vimentin Ser72 and troponin I Ser23/24. These findings establish the AKAP Cypher/ZASP as a signalling hub in the progression of DCM.

## Introduction

Dilated cardiomyopathy (DCM) is a non-ischaemic myocardial disease characterised by ventricular dilatation and systolic dysfunction; it commonly contributes to congestive heart failure and cardiac transplantation (1). Clinically, Cypher/Z-band alternatively spliced PDZ-motif protein (ZASP) mutations are present in various cardiac and skeletal myopathies (2–5), including DCM. Cypher/ZASP is a PDZ-LIM domain protein anchored at the Z-line, with specific expression in skeletal and cardiac muscles (6). Both global and cardiac-specific deletions of Cypher cause severe DCM in animal models; the former exhibits perinatal death within 24 h (7), and the latter displays premature lethality (8). Previous studies have demonstrated that Cypher/ZASP binds to α-actinin-2 and that it is critical for maintaining the Z-line structural stability during contraction (7–9). Several recent reports have linked Cypher/ZASP to signal transduction. For example, Cypher/ZASP cooperates with talin for α5β1-integrin activation (10, 11), and it acts on the protein kinase B-dependent pathway in cardiomyocyte apoptosis (12). However, the molecular mechanisms of DCM induced by Cypher/ZASP deficiency are not well understood, especially with respect to signal transduction.

Cyclic adenosine monophosphate/protein kinase A (cAMP/PKA) signalling, which is activated by β-adrenergic receptors, directs various processes including contractility, metabolism, ion fluxes, hypertrophy, and cell death in cardiomyocytes (13, 14). These processes are closely associated with DCM. PKA-anchoring proteins (AKAPs) are special factors that bind the regulatory subunits of PKA to sequester PKA within subcellular locations spatially and temporally (15). Currently, over 70 AKAPs are recognised, and more than 14 are recognised in the myocardium (14). Our previous work identified Cypher/ZASP as an AKAP, which tethers the type II regulatory subunit RIIα of PKA to modulate the phosphorylation of the L-type calcium channel at Ser1928 (16, 17). Additional specific PKA substrates regulated by AKAP Cypher/ZASP remain unknown. This substantially hinders the understanding of Cypher/ZASP-dependent PKA signalling in the pathogenesis of DCM.

In this study, we conducted a global quantitative phosphoproteomic analysis of heart tissues from wild-type (WT) and Cypher-knockout (KO) mice, treated with isoproterenol for PKA activation. Candidate PKA substrates were selected using motif analysis and further assessed *in vivo* and *in vitro*. We identified β-catenin, vimentin, and troponin I as specific downstream PKA effectors regulated by AKAP Cypher/ZASP.

## Materials and Methods

### Experimental Animals

Global Cypher-KO mice in C57BL/6J background, which have previously been described (7), were a gift from Dr. Ju Chen (Department of Medicine, University of California San Diego, La Jolla, CA). The Cypher-KO mice were generated by breeding Cypher+/– mice, and wild-type mice from the same litter were used as controls. All mice were bred in a pathogen-free environment.

### Mouse Heart Tissue Sample Preparation

Neonatal WT (*n* = 3) and Cypher-KO (*n* = 3) mice were injected intraperitoneally with isoproterenol (15 mg/kg, 30 min) immediately after birth (16). The hearts were harvested, washed with precooled PBS, and quickly frozen in liquid nitrogen. The frozen hearts were mechanically homogenised in lysis buffer containing Halt™ protease phosphatase inhibitors (Thermo Fisher, USA). After centrifugation at 12,000 × g for 20 min at 4°C and protein quantification, 1 mg of protein from the supernatant was collected and reduced to a final concentration of 4.5 mM through the addition of dithiothreitol at 55°C for 30 min. The samples were then alkylated with iodoacetamide (10 mM) at room temperature for 15 min in the dark, followed by trypsin (1:100) digestion for 16 h at 37°C. This reaction was stopped using formic acid (1% in solution).

### Phosphopeptide Enrichment

TiO_2_ spin tips (Thermo Fisher) were used for phosphopeptide enrichment, according to the manufacturer's protocol. Peptides and TiO2 beads (1:4) were mixed in 500 μL of binding buffer and incubated for 30 min at room temperature. The mixture was centrifuged at 2000 × *g* for 2 min, and the supernatant containing mainly non-phosphopeptides was discarded. The beads were transferred into a clean tube and washed with 500 μL of wash buffer more than four times. After the final wash, the beads were centrifuged at 250 × *g* for 10 min for removing the liquid. Bound phosphopeptides were eluted with 15% NH_4_OH/50% ACN and desalted using a C18 column (Waters, USA).

### Liquid Chromatography–Mass Spectrometry (LC-MS)

Peptides were analysed using an Ultimate 3,000 nanoflow liquid chromatography system (Thermo Scientific, USA) coupled with a Q-Exactive HFX mass spectrometer (Thermo Scientific). Gradient elution of 3–80% ACN in 0.1% formic acid was employed. LC-MS data were acquired in a data-dependent mode using the following settings: electrospray voltage of 2.2 kV; automatic gain control target of 3e6 ions at a resolution of 120,000; scan range of 350–1,800 m/z; maximum injection time of 120 ms; isolation window of 1.0 m/z; normalised collision energy of 28; and dynamic exclusion time of 30 s. MS spectra were recorded using the Xcalibur software version 2.3 (Thermo Scientific).

### Quantitative Phosphoproteomic Analysis

Raw data obtained from LC-MS were analysed using the free MaxQuant software (version 1.6.2.0; Max Planck Institute of Biochemistry, Martinsried, Germany), followed by a search against the mouse UniProtKB database. Methionine oxidation and phosphorylation of STY were set up as variable modifications and carbamidomethylation as a fixed modification. Trypsin was selected as the digestion enzyme with no more than two missed cleavages. The tolerances of the first and main search were set at 15 and 4.5 ppm, respectively. A false discovery rate of < 1% was adopted to filter the results. Altered phosphopeptides with fold changes of > 2.0 or < 0.5 were considered statistically significant. Sites with a phosphosite probability above 0.7 were assumed to be truly phosphorylated. Further, standard bioinformatic analysis was performed. In particular, the Database for Annotation, Visualisation, and Integrated Discovery (DAVID, version 6.8; https://david.ncifcrf.gov/) was used to catalogue protein classes and for functional annotation. Signal transduction pathways related to differentially expressed phosphoproteins were identified using the Kyoto Encyclopaedia of Genes and Genomes (KEGG) database (https://www.genome.jp/kegg). Phosphorylation motifs were analysed using WebLogo (http://weblogo.berkeley.edu/). The motif width was 13 and extracted from Ser and Thr sites at position 0.

### Cell Culture

The non-cardiac cell line HEK293T and rat cardiomyocyte cell line H9C2 were purchased from the Cell Bank of the Chinese Academy of Sciences. Cells were cultured in high-glucose DMEM (GENON, China) supplemented with 10% foetal bovine serum (Gibco, USA), penicillin (100 U/mL), and streptomycin (100 U/mL) at 37°C in a humidified atmosphere of 5% CO2. Some groups were stimulated with forskolin (100 μM, 30 min) and 3-isobutyl-1-methylxanthine (IBMX) (250 μM, 30 min) to activate PKA signalling.

### Plasmids and Transfection

Plasmids tagged with Myc or Flag were constructed in the pcdna3.1 (+) vector. Cypher and its PDZ domain deletion mutant (ΔPDZ) were tagged with Myc, while β-catenin and its mutants (S675A and ΔDL), troponin I, and vimentin and its deletion mutant (ΔDDLE) were tagged with Flag. The plasmids were transfected with Lipofectamine 3,000 (Invitrogen, USA) according to the manufacturer's instructions. HEK293T cells were collected 24 h posttransfection for further experiments.

### Small Interfering RNA (SiRNA) Transfection

Small interfering RNA was transfected into H9C2 cells using Lipofectamine RNAiMAX (Invitrogen) by following the manufacturer's instructions. The H9C2 cells were harvested for total protein or RNA extraction 48 h after siRNA treatment. Cypher siRNA and scramble control siRNA were purchased from GenePharma (Shanghai, China). The Cypher target sequence was GGAACAGCCUCUUCCACAUTT.

### Real-Time Quantitative Polymerase Chain Reaction (RT-QPCR)

A Total RNA Mini-preps kit (Sangon Biotech, China) was used to extract RNA from the cells. RNA (1,000 ng) was reverse-transcribed to cDNA using the HiScript®III qRT SuperMix (Vazyme, China). RT-qPCR was performed according to the protocol for the SYBR Green qPCR Master Mix (Vazyme). GAPDH served as an internal reference. All the primer sequences are listed in Supplementary Table 1.

### TOP/FOP Flash Luciferase Reporter Assay

TOP flash (VT8105) and FOP flash (VT8196) plasmids were purchased from YouBio Company. The plasmid with seven lymphoid enhancer factor or T-cell factor (LEF/TCF)-binding sites was named TOP flash, and the plasmid with six mutated LEF/TCF-binding sites was named FOP flash. Cypher or an empty vector with either TOP or FOP flash reporter plasmids was cotransfected into HEK293T cells. The pRL-TK renilla plasmid (Promega, USA) was used as an internal control. After 24 h of incubation, firefly and renilla luciferase activities were measured using a Dual Luciferase Reporter Assay Kit (Vazyme). Firefly luminescence (TOP or FOP flash) was normalised to that of renilla luminescence. LEF/TCF transcriptional activity is presented as the relative TOP/FOP ratio.

### Western Blotting

Mechanically homogenised frozen hearts and fresh cell suspensions were lysed in RIPA buffer (Beyotime, China) with protease and phosphatase inhibitors (Sangon Biotech) on ice for 30 min. Protein concentrations were estimated using BCA assay (Beyotime) and subsequently adjusted to equal levels. Samples (30 μg) were loaded onto polyacrylamide gels and hybridised onto polyvinylidene difluoride membranes (Millipore, USA). Membranes were blocked with 5% BSA for 1 h and probed with the relevant primary antibodies for 16 h at 4°C. After washing, the membranes were incubated at room temperature for 1 h with appropriate secondary antibodies (CST, USA). The blots were developed using ECL (Millipore). The primary antibodies were GAPDH (CST, 5174), Cypher (Abnova, H00011155-M06), β-catenin (Abcam, 32572), β-catenin Ser675 (CST, 4176), β-catenin Ser552 (CST, 9566), β-catenin Ser33/37/Thr41 (CST, 9561), Gsk-3β (CST, 12456), Gsk-3β Ser9 (CST, 5558), vimentin (Abcam, 92547), vimentin Ser72 (Abcam, 52944), stathmin (Abcam, 52630), stathmin Ser16 (Abcam, 47382), troponin I (Abcam, 209809), troponin I Ser23+24 (Abcam, 190697), cyclin D1 (Abcam, 134175), c-Myc (Abcam, 32072), c-jun (Abcam, 32137), Myc (CST, 2276), Flag (Beyotime, AF519), and rabbit IgG (CST, 2729). The ImageJ software was used to quantify the relative protein levels.

### Coimmunoprecipitation (Co-IP)

Heart or cell lysates were divided into input, IP, and IgG groups and incubated with primary antibodies or IgG overnight at 4°C on a roller. Protein A/G magnetic beads (Bio-Rad, USA) were transferred into tubes and washed three times with PBS-T (PBS + 0.1% Tween 20). Subsequently, the lysates were added to the beads and incubated with rotation for 1 h. The beads were washed five times with PBS-T for reducing non-specific binding. Bound proteins were eluted in the loading buffer and boiled for 10 min at 95°C. Elution liquids were removed for western blotting.

### Immunofluorescence and Immunohistochemical Staining

Neonatal mouse hearts were fixed in 4% paraformaldehyde for 48 h, dehydrated with ethanol, and embedded in paraffin. Tissue sections (4 μm) were cut, dewaxed, and rehydrated. Subsequently, antigens were retrieved in a citric acid solution through microwave heating. Afterwards, the samples were permeabilised with 0.2% Triton X-100 at room temperature for 15 min and washed with PBS. After blocking with 5% BSA for 1 h at room temperature, the sections were incubated with diluted primary antibody overnight at 4°C. The sections were washed and incubated with secondary antibodies at room temperature for another hour. Samples were washed again and treated for 10 min with 4–6-diamidino-2-phenylindole (DAPI) (Servicebio, China) or haematoxylin (Servicebio) for labelling the nuclei. Finally, the slides were sealed with antifluorescence quenching and examined using a confocal microscope (Nikon A1). Successive Z-stack sections with 0.15 μm Z-step were examined. The primary antibodies used were as follows: Cypher (Abnova, H00011155-M06), β-catenin (Abcam, 32572), vimentin (Abcam, 92547), integrin β1 (Abcam, 179471), α-actinin (Abcam, 9465), pH3 (CST, 53348), PCNA (Abcam, 29), and Ki-67 (Abcam, 92742).

### Statistical Analysis

The GraphPad Prism software version 9.0 was used for statistical analysis. Normal distribution and homogeneity of variances were assessed using the Kolmogorov–Smirnov test and Bartlett's test. The data are presented as the mean ± SEM. The differences between two groups were analysed using unpaired two-tailed Student's *t*-test. Multiple-group comparisons (two factors) were made using two-way ANOVA, followed by the Bonferroni *post hoc* test. Statistical significance was set at *p* < 0.05.

## Results

### Phosphoproteomic Workflow and Bioinformatic Analysis

**Figure 1:**
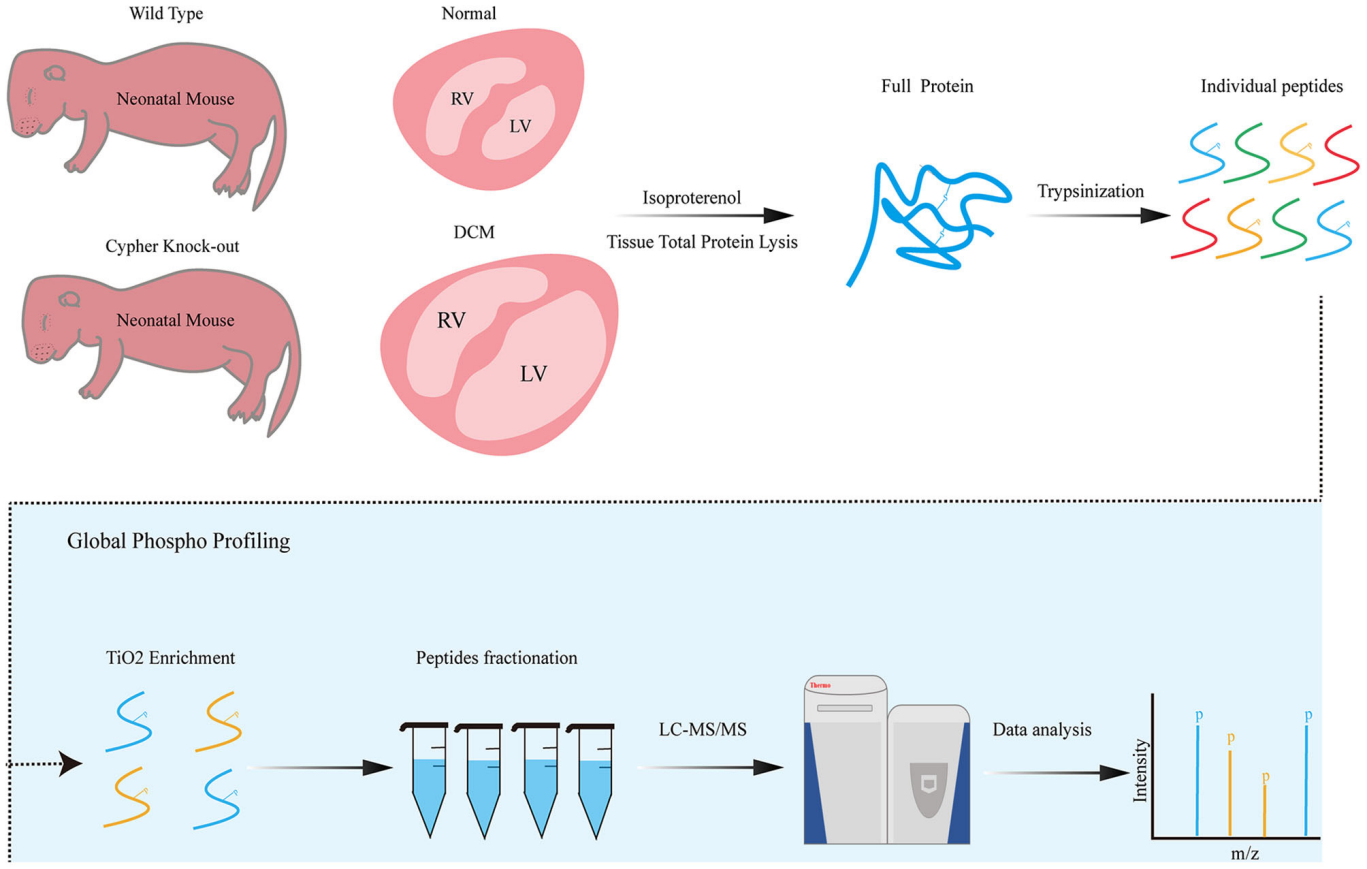
Phosphoproteomics workflow. Neonatal wild-type and Cypher-knockout mice were administered isoproterenol (15 mg/kg, 30 min) before the hearts were harvested. Heart tissue lysates were digested into peptides with trypsin overnight, followed by phosphopeptide enrichment using titanium dioxide spin tips. After washing, bound phosphopeptides were eluted and desalted with alkaline C18 for direct LC-MS analysis. Raw data were analysed using the MaxQuant software. DCM, dilated cardiomyopathy; isoproterenol, a PKA activator; TiO2, titanium dioxide; LC-MS, liquid chromatography–mass spectrometry.

**Figure 2:**
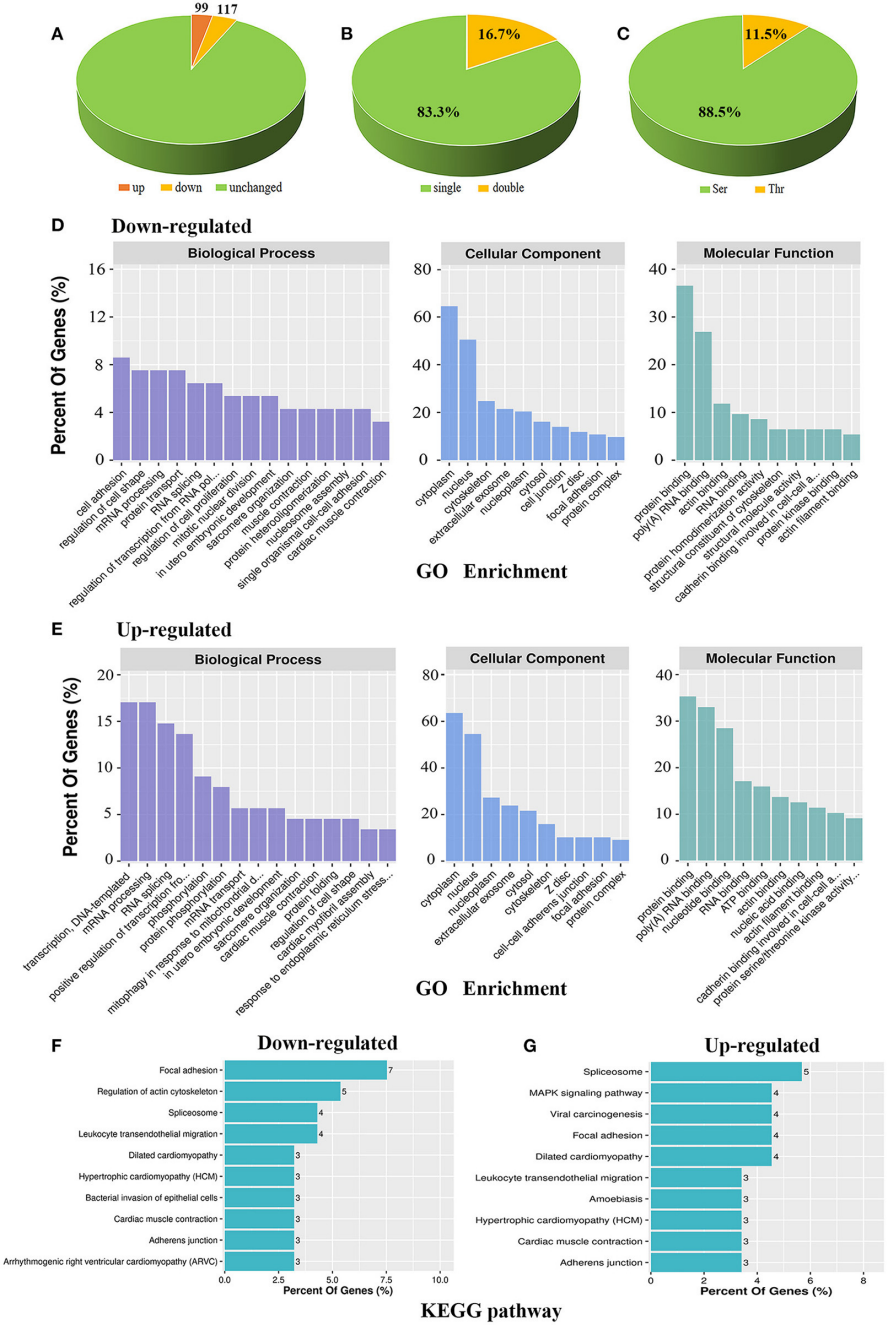
Bioinformatic analysis of the phosphoproteomic results. **(A)** Distribution of differential phosphopeptides in Cypher-knockout (KO) mice compared to that in wild-type (WT) mice. **(B)** Distribution of single and double phosphosites based on differential phosphopeptides. **(C)** Distribution of serine (Ser) and threonine (Thr) phosphorylation based on differential phosphopeptides. **(D,E)** GO enrichment for biological process, cellular component, and molecular function in downregulated **(D)** and upregulated **(E)** phosphoproteins. **(F,G)** KEGG analysis of the top 10 signalling pathways that converge on downregulated **(F)** and upregulated **(G)** phosphoproteins.

We previously found that AKAP Cypher/ZASP could enhance the phosphorylation of L-type calcium channels at Ser1928, especially when the PKA signalling pathway is activated (16). To maximise our chances of identifying specific PKA substrates regulated by AKAP Cypher, newborn WT and Cypher-KO mice were administered the PKA agonist isoproterenol (15 mg/kg, 30 min) before the hearts were harvested. Quantitative phosphoproteomics was performed on the whole heart tissue from the WT and Cypher-KO mice (Figure 1). In total, we acquired 2,887 phosphopeptides corresponding to 1,448 phosphoproteins from the heart tissue. Compared with the WT mice, the Cypher-KO mice presented 216 differentially expressed phosphopeptides, with 99 hyperphosphorylated peptides (fold change > 2.0) and 117 hypophosphorylated peptides (fold change < 0.5) (Figure 2A, Supplementary Tables 2, 3). Among these, 180 peptides (83.3%) were singly phosphorylated (Figure 2B) and 191 peptides (88.5%) were phosphorylated at Ser residues (Figure 2C).

Functional annotations of up- and downregulated phosphoproteins were assessed. Gene ontology (GO) enrichment was annotated with biological process, cellular component, and molecular function. For biological process, downregulated phosphoproteins were enriched mostly in cell adhesion, regulation of cell shape, mRNA processing, and protein transport (Figure 2D, left); meanwhile, upregulated phosphoproteins showed specific enrichment in transcription, DNA-templated, mRNA processing, RNA splicing, and positive regulation of transcription from RNA polymerase II promoter (Figure 2E, left). For cellular component, both up- and downregulated phosphoproteins were mainly distributed in the cytoplasm and nucleus (Figures 2D,E, middle). For molecular function, both hypo- and hyperphosphorylated proteins were mostly involved in protein binding and poly (A) RNA binding (Figures 2D,E, right), while hyperphosphorylated proteins were additionally involved in nucleotide binding (Figure 2E, right). Moreover, KEGG pathway analysis revealed the association of downregulated phosphoproteins with key signalling pathways including focal adhesion, regulation of actin cytoskeleton, spliceosome, and leukocyte transendothelial migration (Figure 2F). In contrast, upregulated phosphoproteins were primarily associated with pathways including spliceosome, MAPK signalling pathway, viral carcinogenesis, focal adhesion, and DCM (Figure 2G). Overall, up- and downregulated phosphoproteins shared certain annotations, such as mRNA processing and focal adhesion, indicating that some differentially expressed protein function synergistically.

### Identification of Phosphorylation-Specific Motifs and Candidate PKA Substrates

**Figure 3:**
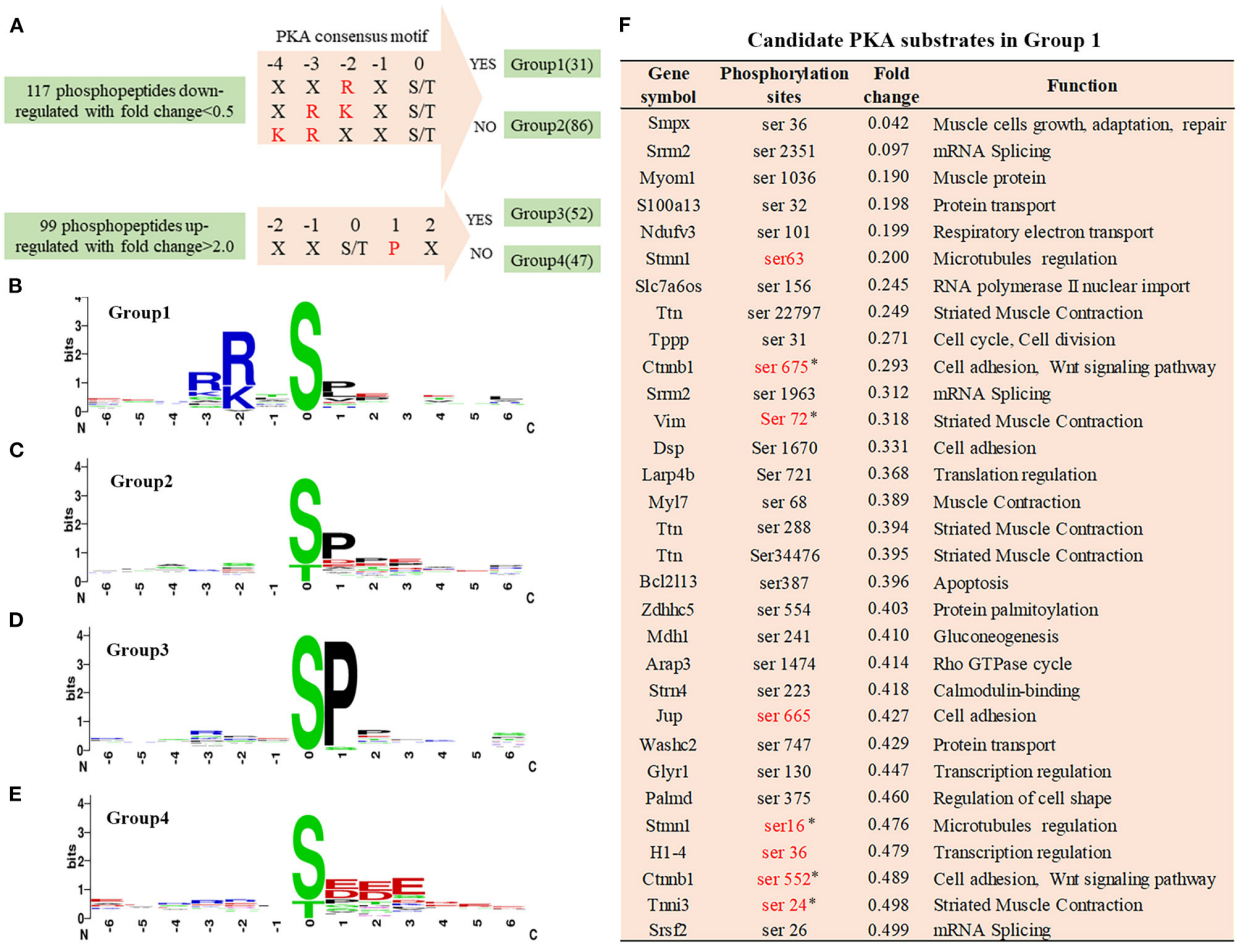
Phosphorylation-specific motif analysis and candidate PKA substrates. **(A)** Grouping of differentially phosphopeptides. The 117 hypophosphorylated peptides in Cypher-KO mice were categorised into two groups: Group 1, containing phosphopeptides with PKA consensus motifs and Group 2, without the motifs. The 99 hyperphosphorylated peptides were divided into the other two groups: Group 3, containing phosphopeptides with SP motif and Group 4, without the motif. **(B–E)** The logo motif of phosphosites in Group 1 **(B)**, Group 2 **(C)**, Group 3 **(D)**, and Group 4 **(E)**. Sequence logos were created using WebLogo. **(F)** The list of candidate PKA substrates regulated by Cypher in Group 1. Sites marked in red are the reported PKA phosphorylation sites. Sites selected for further study are marked with an asterisk (*).

In the Cypher-KO mice, the hypophosphorylated proteins with PKA consensus motifs are likely to be candidate PKA substrates regulated by Cypher. Thus, the 117 downregulated phosphopeptides were aligned and categorised into two groups: Group 1 containing phosphopeptides with the PKA consensus motifs, and Group 2 without the motifs (Figure 3A). Motif analysis of the 99 upregulated phosphopeptides showed a strong predilection for the motif sequence SP (a proline residue at the +1 position) (Figure 3D), based on which Group 3 (with SP motif) and Group 4 (without SP motif) were divided (Figure 3A). The PKA consensus motifs (18) include RXp[S/T], R[R/K]Xp[S/T], or KRXXp[S/T], where X can be any amino acid residue (Figure 3A). Motif analysis showed that the top-enriched motif sequence of peptides in Group 1 (*n* = 31) was RRXS (Figure 3B). The peptides in Group 2 (*n* = 86) and Group 3 (*n* = 52) showed a preference for motif sequence SP (Figures 3C,D), which is a hallmark of glycogen synthase kinase 3 (GSK-3), mitogen-activated protein kinase (MAPK), and cyclin-dependent kinase (CDK) substrates (19). The primary motif sequence enriched in Group 4 (*n* = 47) was S[D/E]XE (Figure 3E) phosphorylated by casein kinase II (CKII) or G-protein-coupled receptor kinase 1 (GRK1) (19).

Motif analysis suggested that the phosphopeptides in Group 1 were direct PKA substrate candidates, which were involved in cell adhesion and muscle contraction (Figure 3F). Eight sites marked in red are the reported PKA phosphorylation sites, and the other 23 sites have not yet been reported. Combined with reference mining, five phosphorylation sites matching four proteins were selected for further validation (Figure 3F): β-catenin Ser675/Ser552, vimentin Ser72, troponin I Ser24, and stathmin Ser16.

### Cypher Interacts With β-Catenin and Facilitates Its Phosphorylation at Ser675 by PKA

**Figure 4:**
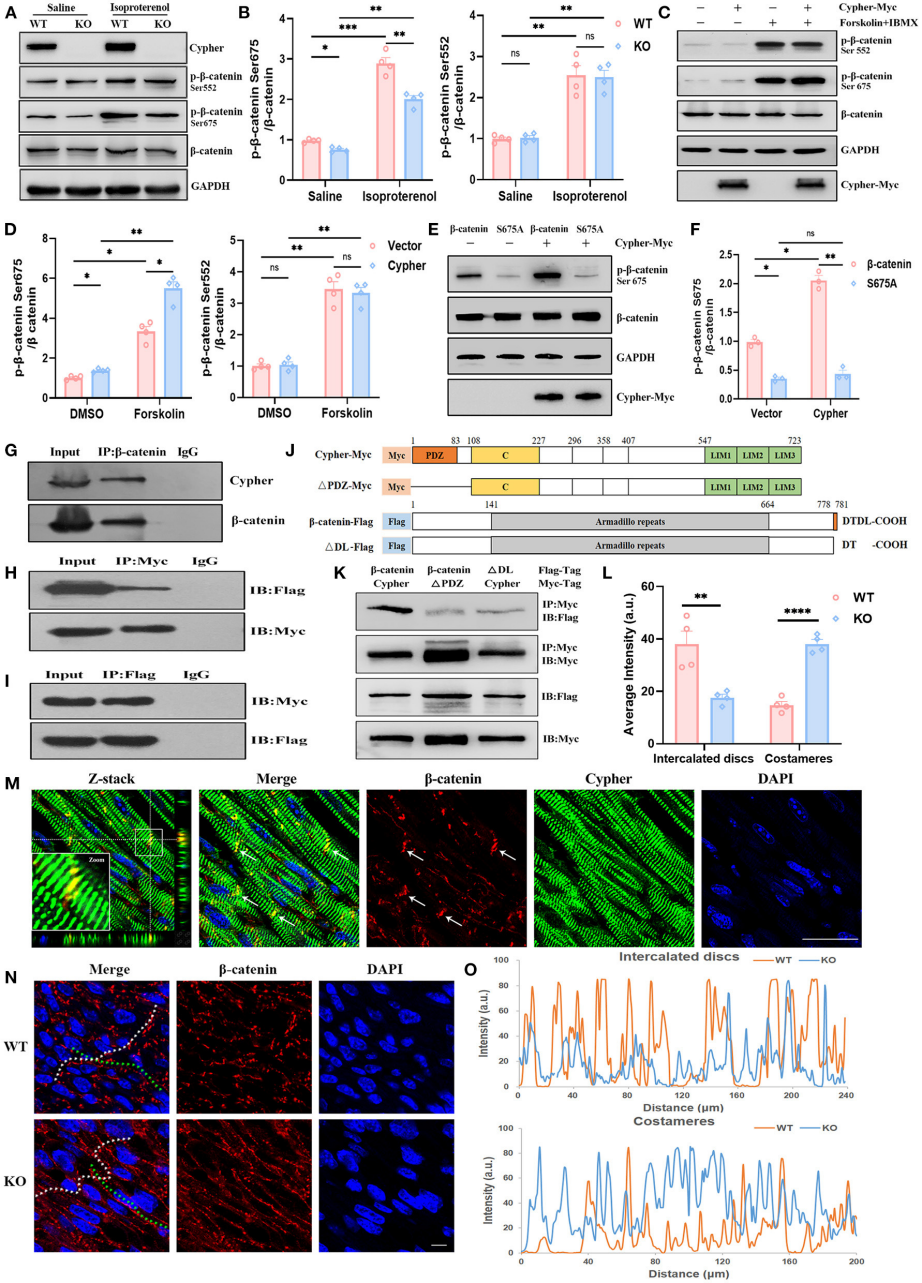
Cypher interacts with β-catenin and facilitates its phosphorylation at Ser675 by PKA. **(A)** Western blotting for β-catenin and its phosphorylation using heart lysates from neonatal WT and Cypher-KO mice. Isoproterenol (15 mg/kg, 30 min) was administered by intraperitoneal infusion before harvesting. **(B)** Quantification of p-β-catenin (Ser675 and Ser552) protein levels in heart lysates. *N* = 4 biologically independent samples. **(C,D)** Western blotting results showing p-β-catenin (Ser675 and Ser552) in Cypher-overexpressing HEK293T or empty vector control cells. The cells were treated with forskolin and IBMX (3-isobutyl-1-methylxanthine) for 30 min before collection. Results of quantitative analysis are shown **(D)**. *N* = 4 biologically independent samples. **(E,F)** β-catenin or its mutant S675A was coexpressed with Cypher in HEK293T cells. The phosphorylated β-catenin at Ser675 with forskolin treatment was assessed and quantified. *N* = 3 biologically independent samples. **(G)** β-catenin interacted with Cypher *in vivo*. β-catenin was immunoprecipitated from neonatal mouse heart lysates, and the interaction with Cypher was validated by anti-Cypher antibody. Rabbit IgG served as the negative control. **(H,I)** β-catenin interacted with Cypher *in vitro*. Myc-tagged Cypher was coexpressed with Flag-tagged β-catenin in HEK293T cells. Immunoprecipitation (IP) was performed with an anti-Myc **(H)** or anti-Flag **(I)** antibody, following which probing was performed using immunoblotting (IB) with an anti-Flag **(H)** or anti-Myc **(I)** antibody. **(J)** Schematic presentation of Myc-tagged Cypher (Cypher-Myc) and its PDZ domain deletion mutant (ΔPDZ-Myc), and also Flag-tagged β-catenin (β-catenin-Flag) and its C-terminal deletion mutant (ΔDL-Flag). C, cardiac specific region. **(K)** Deleting the PDZ domain in Cypher or disrupting the C-terminal PDZ-binding motif (DTDL) in β-catenin significantly weakened the Cypher–β-catenin interaction. Immunoprecipitation of Myc-tagged protein from HEK293T cells and the interacting protein was evaluated using an anti-Flag antibody. **(M)** Immunofluorescence showing Cypher colocalised with β-catenin in the ICD (arrows) in postnatal day-seven (P7) heart sections. The white box indicates the magnified regions. Successive Z-stack sections were acquired at Z-steps of 0.15 μm. DAPI, 4′,6-diamidino-2-phenylindoledihydrochloride. **(N)** Altered distribution of β-catenin in the ICD and costameres when Cypher was absent. The ICD are highlighted with white dashed lines and the costameres with green dashed lines. **(O)** Intensity profiles of β-catenin distribution in the ICD (upper) and costameres (bottom) were measured using the Plot Profile in the ImageJ software. The average intensity was quantified **(L)**. Scale bars: 25 μm. *, *p* < 0.05; **, *p* < 0.01; ***, *p* < 0.001; ****, *p* < 0.0001; ns, no significant difference. Error bars indicate the mean ± SEM. Two-way ANOVA **(B,D,F)** with Bonferroni multiple comparison test and unpaired two-tailed Student's *t*-test **(L)** was used.

Aberrant canonical Wnt/β-catenin signalling is crucial for the pathogenesis of DCM (20–22). Little is known regarding the association between DCM and β-catenin phosphorylation. Typical PKA phosphorylation sites, β-catenin Ser675 and Ser552, showed significantly greater phosphorylation after PKA activation (Figures 4A–D). We analysed heart lysates from neonatal WT and Cypher-KO mice to establish the role of Cypher in β-catenin phosphorylation. Cypher ablation slightly inhibited β-catenin phosphorylation at Ser675 at baseline, and the inhibition became more striking with isoproterenol stimulation (Figures 4A,B). This phenomenon was not observed in the phosphorylation of β-catenin at Ser552 (Figures 4A,B). This effect was subsequently verified in HEK293T cells. We found that β-catenin Ser675 phosphorylation was dramatically elevated with Cypher overexpression and the PKA agonist forskolin stimulation (Figures 4C,D). The elevation was completely blocked by the β-catenin S675A mutation (Figures 4E,F).

Next, we explored whether Cypher affected β-catenin phosphorylation through interaction. *In vivo*, β-catenin was immunoprecipitated from neonatal mouse heart lysate, and Cypher was pulled down together with β-catenin (Figure 4G). In contrast, the rabbit IgG control antibody failed to pull down either protein. The interaction was further verified in HEK293T cells expressing Myc-tagged Cypher and Flag-tagged β-catenin (Figures 4H,I). Analysis of amino acid sequences suggested that β-catenin contains a PDZ-binding motif (DTDL) at its cytosolic tail, and Cypher contains a PDZ domain at its N-terminus (Figure 4J). To ascertain the domain of Cypher that interacts with β-catenin, we constructed a PDZ domain deletion mutant of Cypher (ΔPDZ-Myc) and a deletion mutant lacking the last two residues of β-catenin (ΔDL-Flag) (Figure 4J). Deleting the PDZ domain in Cypher or disrupting the C-terminal PDZ-binding motif (DTDL) in β-catenin significantly weakened the Cypher-β-catenin interaction in HEK293T cells (Figure 4K). Thus, we clarified that Cypher interacted with β-catenin mainly *via* its PDZ domain. Moreover, immunofluorescence demonstrated the colocalisation of Cypher and β-catenin, mainly in the intercalated discs (ICD) of the heart (Figure 4M), where cardiomyocytes connect and communicate (23). Cypher-KO mice displayed a reduced distribution of β-catenin in the ICD, which was accompanied by an increase in lateral borders, namely costameres (Figures 4N,O,L). Cypher thus interacts with β-catenin and facilitates its phosphorylation at Ser675 by PKA.

### Cypher Activates β-Catenin *via* Ser675 Phosphorylation and Modulates Cardiomyocyte Proliferation

**Figure 5:**
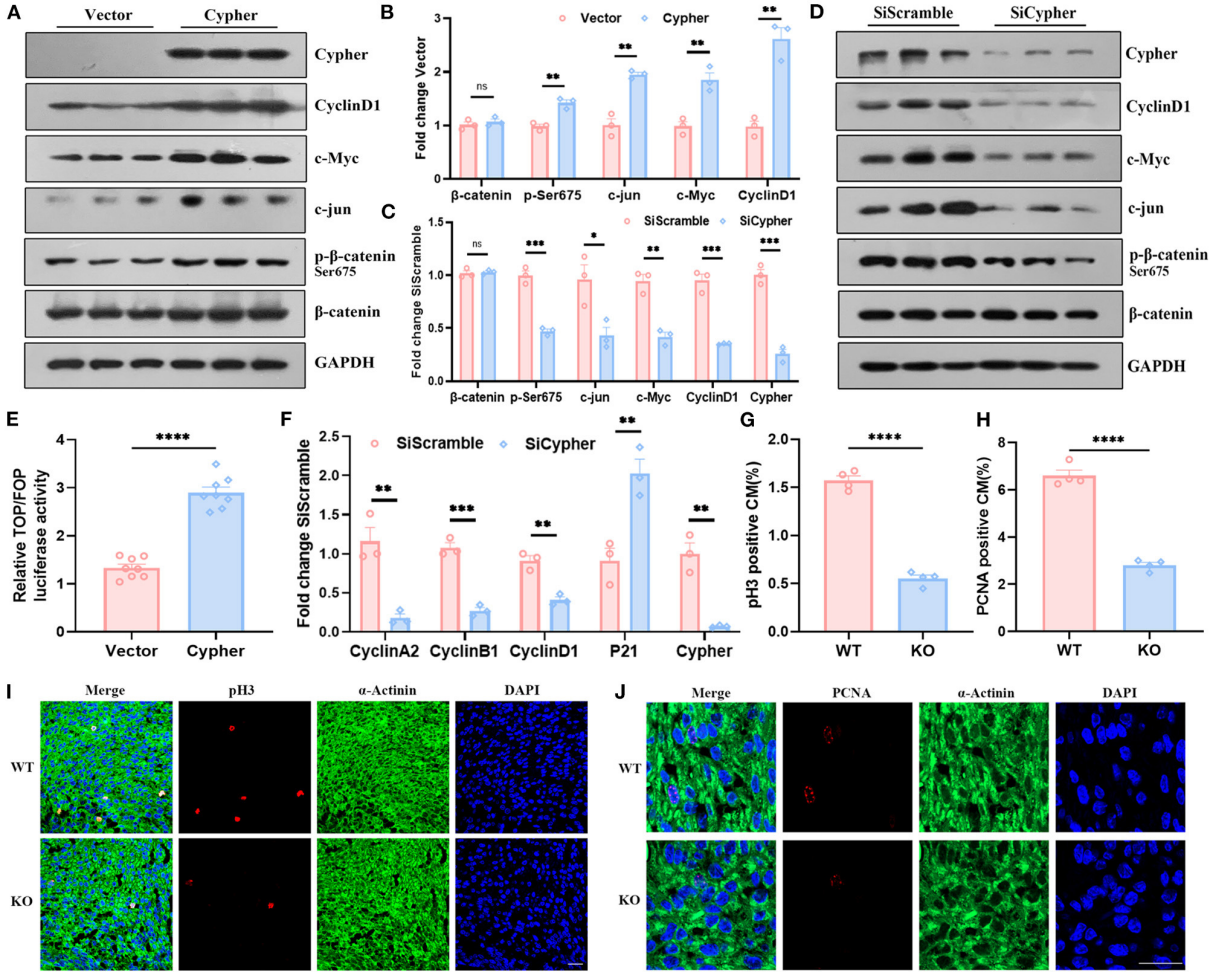
Cypher activates β-catenin *via* Ser675 phosphorylation and modulates cardiomyocyte proliferation. **(A,B)** Myc-tagged Cypher was overexpressed in non-cardiac HEK293T cells with no endogenous Cypher. Empty vector served as a control. The expression of p-β-catenin, β-catenin and its target genes, including cyclin D1, c-Myc, and c-jun, were detected using western blotting and quantified. *N* = 3 biologically independent samples. **(C,D)** H9C2 cells with endogenous Cypher were transfected with scramble control (SiScramble) or Cypher siRNA (SiCypher). Cypher, p-β-catenin, and β-catenin and the abundance of its target genes were examined and quantified. *N* = 3 biologically independent samples. **(E)** Dual-luciferase reporter assays were performed to evaluate β-catenin transcriptional activity. HEK293T cells were cotransfected with Cypher or empty vector and TOP or FOP plasmid. The renilla plasmid was used as an internal control. *N* = 8 biologically independent samples. **(F)** The mRNA levels of the cell cycle-related genes (cyclin A2, cyclin B1, cyclin D1, and P21) were measured in Cypher-knockdown H9C2 cells using real-time quantitative polymerase chain reaction (RT-qPCR). *N* = 3 biologically independent samples. **(G,I)** Immunofluorescence staining of pH3 in the hearts of newborn (P0) WT and Cypher-KO mice. α-actinin+pH3+ labels proliferating cardiomyocytes. **(G)** Quantification of pH3-positive cardiomyocytes in the P0 hearts (6,379 cardiomyocytes in the WT group and 5,773 cardiomyocytes in the Cypher-KO group). *N* = 4 biologically independent samples. **(H,J)** Immunofluorescence staining of PCNA in the P0 WT and Cypher-KO hearts. α-actinin+PCNA+ labels proliferating cardiomyocytes. **(H)** Quantification of PCNA-positive cardiomyocytes in the P0 hearts (4,518 cardiomyocytes in the WT group and 3,587 cardiomyocytes in the Cypher-KO group). *N* = 4 biologically independent samples. Scale bars: 25 μm. *, *p* < 0.05; **, *p* < 0.01; ***, *p* < 0.001; ****, *p* < 0.0001; ns, no significant difference. Error bars indicate the mean ± SEM. Unpaired two-tailed Student's *t*-test **(B,C,E–H)** was used.

Phosphorylation of β-catenin at Ser675 is associated with its transcriptional activity (24–27). To assess β-catenin transcriptional activity, we performed western blotting for its target gene expression and lymphoid enhancer factor or T-cell factor (LEF/TCF) dual-luciferase reporter assays. The levels of β-catenin target genes, including cyclin D1, c-Myc, and c-jun, strikingly increased with Cypher overexpression, which was accompanied by an increase in Ser675 phosphorylation (Figures 5A,B). The opposite effects were observed with Cypher knockdown (Figures 5C,D). Neither Cypher overexpression nor knockdown changed the levels of β-catenin (Figures 5A–D). The phosphorylation of Gsk3β Ser9 and β-catenin Ser33/37/Thr41, which led to β-catenin degradation through the proteasome (28), was not altered in Cypher knockdown H9C2 cells (Supplementary Figures 1A–C). Furthermore, dual-luciferase reporter assays were performed on Cypher-overexpressing and control cells. Relative luciferase activity significantly increased with Cypher overexpression (Figure 5E). All the evidence above elucidated that Cypher is capable of activating β-catenin *via* Ser675 phosphorylation.

Activated β-catenin is closely linked to cell proliferation (24, 29). To explore the role of Cypher in cardiomyocyte proliferation, RT-qPCR for cell cycle-related genes was performed on both untreated and Cypher siRNA-treated H9C2 cells. Cypher knockdown suppressed the mRNA expression of cell cycle-positive regulators, such as cyclin A2, cyclin B1, and cyclin D1, and elevated the expression of the negative regulator P21 (Figure 5F). Meanwhile, immunofluorescence staining illustrated that cardiomyocyte proliferation (labelled α-actinin+pH3+ or α-actinin+PCNA+) was impaired in newborn (P0) Cypher-KO mice (Figures 5G–J) and foetal mice at embryonic day 15 (E15) (Supplementary Figures 1H,I). Immunohistochemical staining for Ki-67 yielded similar results (Supplementary Figures 1D–G). In summary, we demonstrated that Cypher activates β-catenin *via* Ser675 phosphorylation and modulates cardiomyocyte proliferation.

### Cypher Enhances Vimentin Ser72 Phosphorylation by PKA

**Figure 6:**
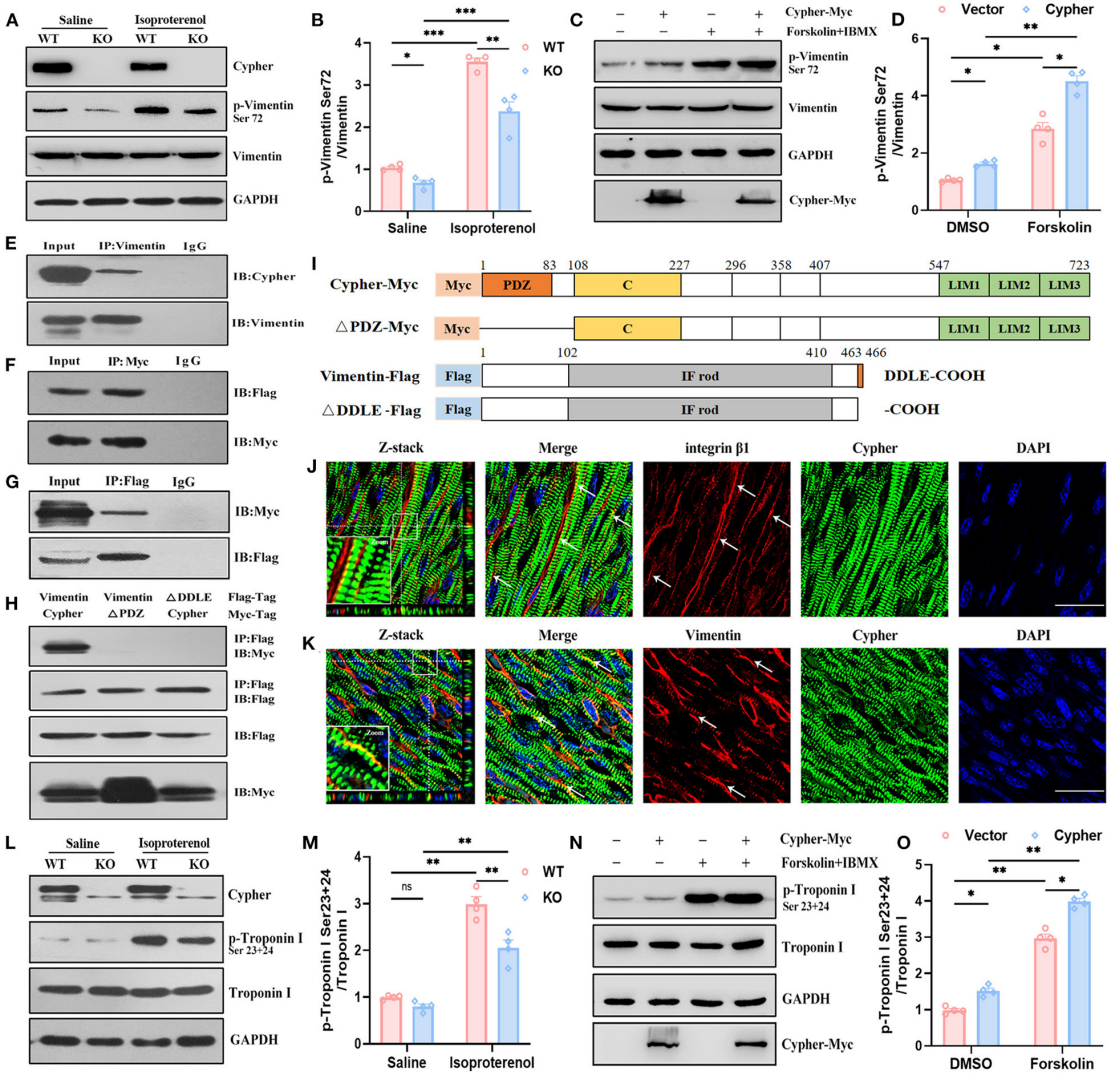
Validation of vimentin and troponin I as PKA substrates regulated by Cypher. **(A,B)** Heart lysates from neonatal WT and Cypher-KO mice were analysed for total and phosphorylated vimentin. The mice were administered isoproterenol (15 mg/kg, 30 min) prior to sacrifice. Quantitative p-vimentin (Ser72) are shown **(B)**. *N* = 4 biologically independent samples. **(C,D)** HEK293T cell lysates with Cypher overexpression were analysed for total and phosphorylated vimentin. Forskolin (100 μM, 30 min) and IBMX were used for activating PKA. p-Vimentin (Ser72) was quantitatively measured **(D)**. *N* = 4 biologically independent samples. **(E)** Vimentin interacted with Cypher *in vivo*. Vimentin was enriched from neonatal mouse heart lysates, and interaction with Cypher was verified using anti-Cypher antibody. Rabbit IgG served as the negative control. **(F,G)** Coimmunoprecipitation (Co-IP) and reverse Co-IP experiments were performed to test the interaction between Cypher and vimentin in HEK293T cells. **(F)** Myc-tagged Cypher was enriched using anti-Myc antibody, and the interacting protein was probed with anti-Flag antibody. **(G)** Flag-tagged vimentin was enriched using anti-Flag antibody, and the interacting protein was analysed using anti-Myc antibody. **(H)** The Cypher PDZ domain interacted with the vimentin C-terminal PDZ-binding motif (DDLE). Myc-tagged Cypher or its PDZ domain deletion mutant (ΔPDZ-Myc) and Flag-tagged vimentin or its deletion mutant (ΔDDLE-Flag) were cotransfected into HEK293T cells. Immunoprecipitation was performed using anti-Flag antibody, and the samples were further probed with anti-Myc antibody. **(I)** Schematic presentation of Myc-tagged and Flag-tagged proteins mentioned in **(H)**. C, cardiac specific region; IF rod, intermediate filament rod domain. **(J)** Colocalisation (arrows) of Cypher and integrin β1 indicated that Cypher partially localised to costameres. The white box indicates the magnified regions. Z-stack images with 0.15 μm Z-steps were imaged from neonatal heart sections. **(K)** Immunofluorescence confocal images illustrated the colocalisation of Cypher and vimentin in costameres, as indicated by arrows. The white box indicates the magnified regions. Scale bars: 25 μm. **(L)** Troponin I and its phosphorylation using neonatal heart lysates were detected by western blotting. Isoproterenol (15 mg/kg, 30 min) was administered before the hearts were collected. **(M)** Quantification of p-troponin I (Ser23+24) protein levels in heart lysates. *N* = 4 biologically independent samples. **(N)** Myc-tagged Cypher and Flag-tagged troponin I were coexpressed in HEK293T cells with forskolin (100 μM, 30 min) for activating PKA. Total and phosphorylated troponin I were analysed by western blotting. **(O)** Quantification of p-troponin I (Ser23+24) protein levels in cell lysates. *N* = 4 biologically independent samples. *, *p* < 0.05;**, *p* < 0.01; ***, *p* < 0.001. Error bars indicate the mean ± SEM. Two-way ANOVA **(B,D,M,O)** with Bonferroni multiple comparison test was applied.

Vimentin is commonly recognised as a marker of activated myofibroblasts involved in cardiomyopathy (30), and several studies have uncovered its cardioprotective effects in cardiomyocytes (31, 32). Vimentin and its phosphorylation deserve further exploration. Induction of PKA signalling by isoproterenol *in vivo* and by forskolin *in vitro* resulted in increased vimentin Ser72 phosphorylation, demonstrating that vimentin Ser72 is a PKA phosphorylation site (Figures 6A,C). In the absence of Cypher, vimentin Ser72 phosphorylation was reduced both at basic levels and following isoproterenol administration (Figures 6A,B). Furthermore, vimentin Ser72 phosphorylation was greatly elevated in cells overexpressing Cypher following forskolin treatment. This effect was blunted in Cypher-null cells (Figures 6C,D). Thus, vimentin Ser72 is a PKA phosphorylation site promoted by Cypher.

To determine whether Cypher promotes vimentin Ser72 phosphorylation by direct interaction, immunoprecipitation experiments were performed in heart lysates from neonatal mice. The experiments demonstrated the interaction of vimentin with endogenous Cypher (Figure 6E). Furthermore, the binding was confirmed by coimmunoprecipitation (Co-IP) and reverse Co-IP experiments in HEK293T cells (Figures 6F,G). In addition, vimentin was predicted to contain a PDZ-binding motif (DDLE) at its cytosolic tail (Figure 6I). To investigate the interaction domain, we cotransfected Myc-tagged Cypher or its PDZ domain deletion mutant (ΔPDZ-Myc) and Flag-tagged vimentin or its deletion mutant (ΔDDLE-Flag) into HEK293T cells (Figures 6H,I). Full-length Cypher bound to full-length vimentin (Figure 6H), while neither deletion mutant (ΔPDZ or ΔDDLE) was able to bind. Thus, the PDZ domain of Cypher interacts with the vimentin C-terminal PDZ-binding motif (DDLE).

Furthermore, Cypher was colocalised with integrin β1 (Figure 6J), a marker for submembranous structures, named costameres (33). This revealed that Cypher was partially localised to the costameres. Immunofluorescence confocal images showed colocalisation of Cypher and vimentin at the costameres (Figure 6K). However, the distribution of vimentin was not altered by the Cypher deletion (Supplementary Figure 2A).

### Identification of Stathmin and Troponin I as PKA Substrates Regulated by Cypher

Stathmin phosphorylation at Ser16 or Ser63 modulates microtubule dynamics in processes such as proliferation and migration (34, 35). Cardiac troponin I (cTnI) is an important sarcomeric component responsible for cardiac contraction and relaxation (36). PKA-mediated phosphorylation of cTnI Ser23/24 is crucial for myofilament Ca^2+^ sensitivity and cardiac function (37). The expressions of stathmin, cTnI, and their phosphorylated forms in neonatal heart lysates from WT and Cypher-KO mice were determined by western blotting. Isoproterenol stimulation induced evident phosphorylation of stathmin Ser16 and cTnI Ser23/24 (Supplementary Figures 2B,C, Figures 6L,M). Cypher deletion did not alter stathmin Ser16 phosphorylation with or without isoproterenol (Supplementary Figures 2B,C), whereas isoproterenol stimulation dramatically enhanced the phosphorylation of cTnI at Ser23/24 in the WT hearts. This effect was blunted in the hearts with Cypher ablation (Figures 6L,M). In HEK293T cells with Myc-tagged Cypher and Flag-tagged cTnI cotransfection, Cypher overexpression obviously increased cTnI Ser23/24 phosphorylation, especially in PKA activation (Figures 6N,O). However, Co-IP experiments showed that Cypher failed to pull down cTnI in cells (Supplementary Figure 2D), and cTnI failed to pull down Cypher endogenously or exogenously (Supplementary Figures 2E,F). Thus, it appears highly likely that Cypher indirectly affects the phosphorylation of cTnI.

## Discussion

Through a global quantitative phosphoproteomic analysis, we identified 31 candidate PKA phosphorylation sites. Among them, β-catenin Ser675 phosphorylation was validated to be promoted by Cypher, which could enhance β-catenin transcriptional activity and modulate cardiomyocyte proliferation. Moreover, the phosphorylation of two other PKA substrates, vimentin Ser72 and troponin I Ser23/24, was facilitated by Cypher.

Cyclic adenosine monophosphate or protein kinase A signalling is critical in the pathophysiology of DCM (13, 14, 38). Dysregulation of PKA-mediated phosphorylation is involved in cardiomyopathy (39). Through quantitative phosphoproteomics, we identified 216 differentially phosphorylated peptides in the absence of Cypher. We aimed to identify the downstream PKA effectors, especially those directly regulated by AKAP Cypher; thus, PKA consensus motifs were used to shortlist the PKA effector candidates (Figure 2A, Group 1). A number of peptides without PKA consensus motifs showed a considerable difference between WT and Cypher-KO mice, possibly because of the following reasons. First, Cypher/ZASP binds to protein kinase B, protein kinase C, and phosphatase calcineurin (12, 16, 40). The phosphorylation of the corresponding substrates has a large probability of being affected by Cypher deficiency, such as serine or threonine-protein kinase D2, and E3 ubiquitin-protein ligase NEDD4 (Supplementary Table 2). Second, these proteins are more likely to be affected by PKA/Cypher-dependent signalling indirectly, as several among them are active in the nucleus and involved in transcriptional regulation (Figure 2E). Moreover, sequence analysis showed a strong predilection for motif sequence SP in Groups 2 and 3 (Figures 3C,D), a hallmark of GSK-3, MAPK, and CDK substrates (19). Accumulating evidence suggests that MAPKs play a key role in the development of DCM (41, 42). The phosphorylation of MAPKs, such as ERK, P38, and MEK3/6, has been shown to be dramatically altered in Cypher-KO mice (8), which strongly supports the efficiency of our proteomic analysis. Additionally, the motif sequence enriched mostly in Group 4 was S[D/E]XE, indicating that CKII or GRK1 (19) might participate in the progression of DCM. In summary, the differentially phosphorylated proteins and kinases listed above function together in the pathogenesis of DCM.

From the 31 candidates in Group 1, β-catenin Ser675, vimentin Ser72, and troponin I Ser24 were verified as specific PKA/Cypher signalling effectors. β-Catenin serves as a structural component of adherens junctions, and it is the main nuclear mediator of canonical Wnt signalling (43). Aberrant Wnt-β-catenin signalling contributes to various cardiac diseases, including DCM (20–22). The canonical Wnt pathway regulates β-catenin signalling by altering its stability (44). In the absence of a Wnt signal, β-catenin is phosphorylated by Gsk3 at Ser33/37/Thr41 and by casein kinase 1 at Ser45 (28, 44). Subsequently, β-catenin is degraded by the ubiquitin–proteasome, resulting in reduced nuclear accumulation (45). In contrast, Wnt ligand stimulation inhibits Gsk3-mediated β-catenin phosphorylation and stabilises β-catenin to activate Wnt target gene expression (24). Recent studies on cAMP/PKA-mediated β-catenin phosphorylation have demonstrated a Wnt-independent mechanism modulating β-catenin signalling (25, 46, 47). In particular, the phosphorylation of β-catenin at Ser675 by PKA promotes its transcriptional activity, which may not affect Gsk3-dependent phosphorylation of β-catenin and its stability (25, 27, 46, 48). Here, we identified β-catenin as a PKA substrate modified by AKAP Cypher (Figures 4A–F) and validated Cypher as a positive regulator of β-catenin transcriptional activity *via* Ser675 phosphorylation (Figures 5A–E). The regulation was not dependent on total β-catenin or Gsk3-dependent phosphorylation (Supplementary Figures 1A–C). This implies that Cypher may activate β-catenin *via* a Wnt-independent pathway. Meanwhile, the expression of Wnt target genes, including cyclin D1, c-Myc, and c-jun, and the activity of Wnt signalling were upregulated by Cypher (Figures 5A–E), indicating that Cypher is related to Wnt signalling. Therefore, it is likely that Cypher-mediated β-catenin Ser675 phosphorylation may serve as a crosstalk between Wnt signalling and the cAMP/PKA pathways.

Activated β-catenin promotes biological processes such as proliferation, angiogenesis, metastasis, and apoptosis inhibition (24–26). Phosphorylation of β-catenin at Ser675 was reported to activate β-catenin and enhance cardiomyocyte proliferation (29). We observed that cardiomyocyte proliferation was suppressed by Cypher deficiency (Figures 5F–J), which is consistent with the “regulation of cell proliferation” enrichment in GO annotation for downregulated proteins (Figure 2D). We previously reported that Cypher/ZASP deficiency induced apoptosis, with a striking decrease in cell viability (12). Recent studies have linked other DCM-associated proteins, such as lamin and Xinβ, to cardiomyocyte proliferation (49, 50). Moreover, Cypher interacted with β-catenin mainly through the PDZ domain (Figures 4G–K), thereby aligning with the finding that the PDZ domain of Shank3 is responsible for its interaction with β-catenin (51). In addition, Cypher colocalised with β-catenin predominantly in ICD, the signalling hubs for pathways including Wnt/β-catenin, p38 MAPK cascade, calcineurin or nuclear factor of activated T-cell signalling, and the Hippo kinase cascade (23). Interestingly, Cypher ablation reduced the distribution of β-catenin in ICD, which might cause adherens junction abnormalities. Adherens junctions are crucial for structural support and mechanical sensing, both of which are closely linked to arrhythmogenic cardiomyopathy (23). These findings suggest avenues for further research on arrhythmogenic cardiomyopathy caused by Cypher/ZASP mutations. Thus, we presume that Cypher participates in cardiomyocyte proliferation by enhancing β-catenin Ser675 phosphorylation and its transcriptional activity during the progression of DCM.

Vimentin, a type III intermediate filament protein, is widely distributed in most cells (30) and has been reported to have a cardioprotective effect in cardiomyocytes (31, 32). Vimentin filament assembly and maturation are modified by phosphorylation at Ser72 (52, 53). Furthermore, vimentin phosphorylation is related to β1-integrin activation through direct interactions (53, 54). In our study, PKA-dependent vimentin Ser72 phosphorylation was promoted by Cypher through interaction (Figures 6A–H), which relies on the PDZ domain of Cypher and the vimentin C-terminal PDZ-binding motif (DDLE). In addition, the colocalisation of Cypher and vimentin at costameres (Figure 6K) suggests that vimentin is incorporated into costameres and that it might cooperate with Cypher. Costameres are Z-line-associated structures that link sarcomeres to the sarcolemma. These structures sense mechanical forces and transduce them into biochemical signals (33, 55). Considering previous evidence that ZASP/Cypher is required for β1-integrin activation (10, 11), we propose that Cypher might cooperate with vimentin in integrin activation. The relevant biological functions linked to integrin activation in cardiomyocytes should be further explored.

Cardiac troponin I, the inhibitory subunit of the troponin complex, is a thin filament regulatory component responsible for cardiac contraction and relaxation (36). The phosphorylation of cTnI is integral to contractile function (37, 56, 57). In particular, classical Ser23/24 phosphorylation mediated by PKA is closely related to myofilament Ca2+ sensitivity and accelerated relaxation (37, 57). We observed that Cypher promoted cTnI Ser23/24 phosphorylation with PKA activation (Figures 6L–O), but no direct interaction was identified. However, Cypher was predicted to interact with cTnI in the STRING database, probably because the Co-IP assay is unable to distinguish weak and transient interactions or because Cypher acts on cTnI indirectly. In either case, cTnI Ser23/24 phosphorylation was suppressed in the absence of Cypher, which in turn caused impaired cardiac relaxation and worsened cardiac performance.

Moreover, we previously reported that the cardiomyopathy-linked Cypher/ZASP mutation T203I altered its interaction with PKA (16). We speculate that the T203I mutation may affect the phosphorylation of these downstream PKA effectors and thus contribute to the DCM phenotype. Available evidence highlights PKA/Cypher as a signalling centre associated with DCM and offers further cues to specific molecular mechanisms and novel therapeutic or diagnostic targets.

This study had several limitations. First, PKA consensus motifs were adopted to select the most likely PKA effectors. This approach probably missed the substrates without the motif. Second, several cardiomyopathy-associated proteins, such as γ-catenin, titin, small muscular protein, and myomesin (58–60), have been identified in the phosphoproteomics data. Further research can be conducted on their function in DCM.

In conclusion, AKAP Cypher is crucial for β-catenin transcriptional activity and cardiomyocyte proliferation *via* β-catenin Ser675 phosphorylation. Additionally, vimentin Ser72 and troponin I Ser23/24 were verified as downstream effectors of PKA/Cypher. Available evidence highlights Cypher as a signalling hub in the pathogenesis of DCM.

## Data Availability Statement

The mass spectrometry proteomics data have been deposited to the ProteomeXchange Consortium (http://proteomecentral.proteomexchange.org) via the iProX partner repository with the dataset identifier PXD029026. The data can also be accessed from iPoX. (https://www.iprox.cn/) with the dataset identifier IPX0003348001.

## Ethics Statement

The animal study was reviewed and approved by Ethics Committee of the First Affiliated Hospital, Zhejiang University School of Medicine.

## Author Contributions

JL and ZP designed all experiments, conducted sample preparation, data analysis, and validation, drew the figures, and wrote the paper. JC and DW performed the phosphoproteomics assay and bioinformatic analysis with the help of JJ. JH, YD, and RX contributed to the coimmunoprecipitation and immunostaining experiments. RX and XY provided expertise in statistical analysis. HC and XG supervised the research and revised the paper. All authors reviewed and approved the submitted version of the manuscript.

## Funding

This work was supported by grants from the National Natural Science Foundation of China (81470370 and 82170331), the Fundamental Research Funds for the Zhejiang Province Universities (2019XZZX003-15), and Key Research and Development Plan of Zhejiang Province (2020C03017).

## Conflict of Interest

The authors declare that the research was conducted in the absence of any commercial or financial relationships that could be construed as a potential conflict of interest.

## Publisher's Note

All claims expressed in this article are solely those of the authors and do not necessarily represent those of their affiliated organizations, or those of the publisher, the editors and the reviewers. Any product that may be evaluated in this article, or claim that may be made by its manufacturer, is not guaranteed or endorsed by the publisher.
