# Supplementary material for: Phosphoproteomic Analysis Reveals Downstream PKA Effectors of AKAP Cypher/ZASP in the Pathogenesis of Dilated Cardiomyopathy
Source: Front Cardiovasc Med. 2021 Dec 13;8:753072. doi: 10.3389/fcvm.2021.753072 (PMC8710605; doi:10.3389/fcvm.2021.753072)
Supplement: Supplementary file 2 [file Data_Sheet_1.docx]

Supplementary Material

# Figure S1


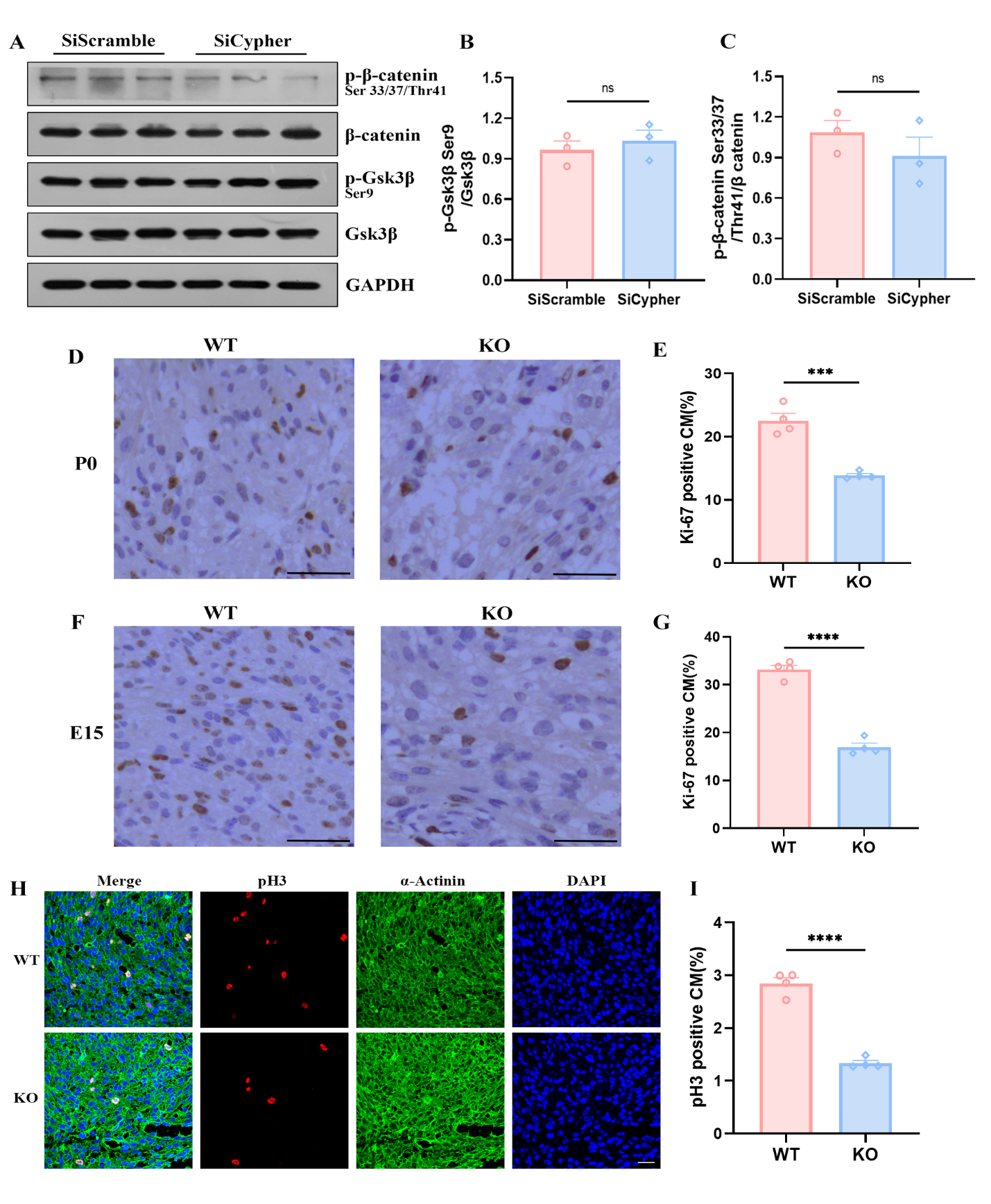


**Figure S1. Cypher ablation inhibites cardiomyocyte proliferation. (A–C)** Western blotting for β-catenin, p-β-catenin (Ser33/37/Thr41), Gsk3β, and p-Gsk3β (Ser9) in Cypher knock-down H9C2 cells. Results of quantitative analysis are shown (B) and (C). N = 3 biologically independent samples. **(D)** Immunohistochemical staining for Ki-67 in newborn (P0) WT and Cypher-KO hearts. **(E)** Quantification of Ki-67 positive cardiomyocytes in newborn hearts (5367 cardiomyocytes in the WT group and 2348 cardiomyocytes in the Cypher-KO group). N = 4 biologically independent samples. **(F)** Immunohistochemical staining for Ki-67 in embryonic day 15 (E15) WT and Cypher-KO hearts. **(G)** Quantification of Ki-67 positive cardiomyocytes in embryonic hearts (3336 cardiomyocytes in the WT group and 4765 cardiomyocytes in the Cypher-KO group). N = 4 biologically independent samples. **(H)** pH3 staining of hearts from E15 WT and Cypher-KO mice. (**I**) Quantification of pH3 positive cardiomyocytes in E15 hearts (4886 cardiomyocytes in the WT group and 4765 cardiomyocytes in the Cypher-KO group). N = 4 biologically independent samples. Scale bars: 25 *μm.* ***, P < 0.001. ****, P < 0.0001. ns, no significant difference. Error bars indicate the mean ± SEM. Unpaired two-tailed Student’s t test (B, C, E, G, I) was used.

# Figure S2


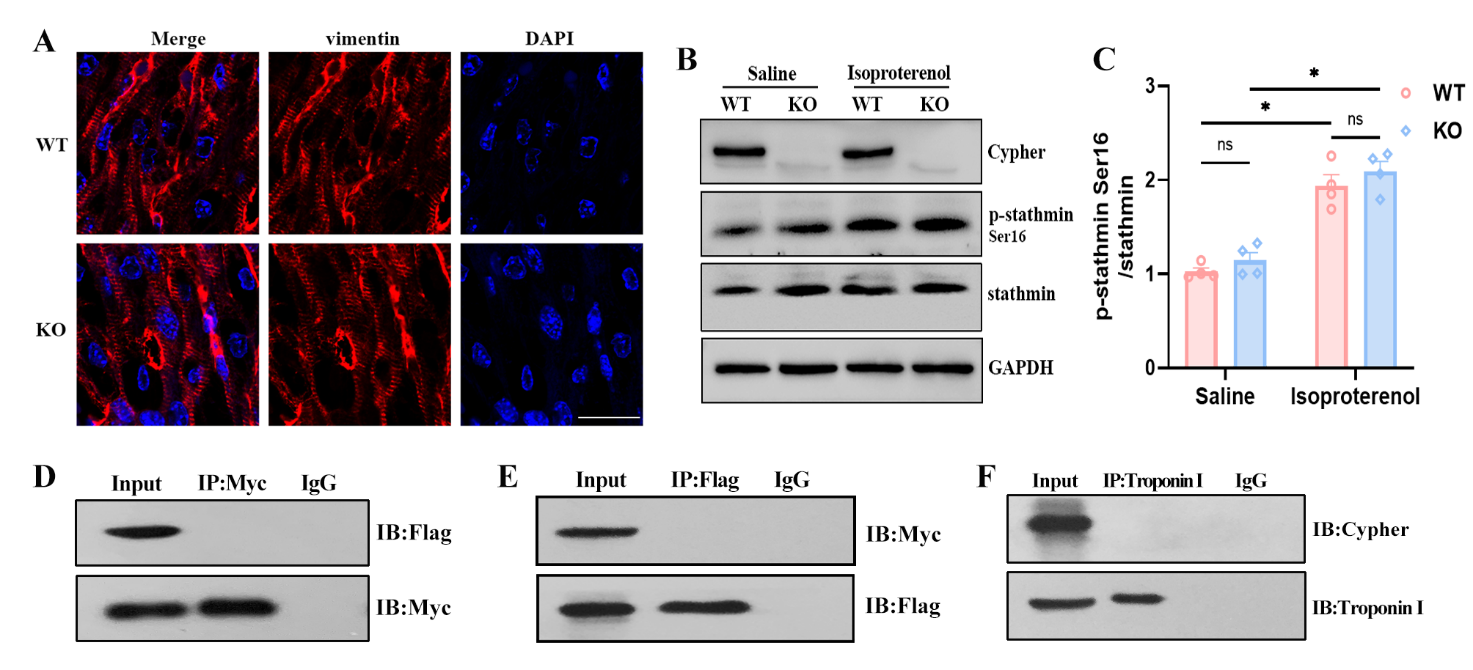


**Figure S2.** **Identification of vimentin, stathmin and troponin I as downstream effectors of PKA/Cypher signalling. (A)** Immunofluorescence staining showed that the distribution of vimentin was not altered in Cypher-KO mice. Scale bars: 25 μm. **(B–C)** The phosphorylation of stathmin (Ser 16) was not affected by Cypher. Heart lysates from neonatal WT and Cypher-KO mice were analysed for total and phosphorylated stathmin. N = 4 biologically independent samples. **(D–E)** Cypher affected the phosphorylation of troponin I indirectly because no interaction was detected. **(D)** Myc-tagged Cypher was enriched by anti-Myc antibody, no Flag-tagged protein was probed. **(E)** Flag-tagged Troponin I was enriched by anti-Flag antibody, no Myc-tagged protein was detected. **(F)** Troponin I was immunoprecipitated from neonatal mouse heart lysate, while Cypher was not detected as the interaction protein. Rabbit IgG served as the negative control. IP, immunoprecipitation; IB, immunoblotting. *, P < 0.05. ns, no significant difference. Error bars indicate the mean ± SEM. Two-Way ANOVA with Bonferroni multiple comparison test (C) was applied.

# Table S1. List of primer sequences for RT-qPCR.

| Gene | Forward (5’-3’) | Reverse (5’-3’) |
| --- | --- | --- |
| CyclinA2 | CAGCCAGACATCACTAACAGTATGC | TCTTGGAATAGGTATCGTCCGTAA |
| CyclinB1 | GCGTAAAGTCAGCGAACAGTCAAG | GCGGAGAGGGAGTATCAACCAAA |
| CyclinD1 | AGCAGAAGTGCGAAGAGGAGGT | GGGCGGATAGAGTTGTCAGTGTAG |
| P21 | GCAAAGTATGCCGTCGTCTGT | GTCAAAGTTCCACCGTTCTCG |
| Cypher | TGACCAGGCAGCAAGGAAC | GACTCCATCAATGGCCACCA |
| GAPDH | AGTGCCAGCCTCGTCTCATA | GATGGTGATGGGTTTCCCGT |

# Table S2. List of up-regulated phosphopeptides (fold change > 2.0), as per label-free mass spectrometry in Cypher-knockout mice.

| **Phosphopeptide sequence with phosphosite probability** | **Leading razor protein** | **fold change** | **Rank** |
| --- | --- | --- | --- |
| IHPEASHPAAIQQDS(1)CEER | sp\|Q3UW53\|NIBAN_MOUSE | 29.191 | 1 |
| RLS(0.914)S(0.063)T(0.017)S(0.005)LAS(0.001)GHSVR | sp\|Q8BZ03\|KPCD2_MOUSE | 20.291 | 2 |
| RRDS(1)DGVDGFEAEGK | sp\|O88532\|ZFR_MOUSE | 19.378 | 3 |
| S(0.002)PS(0.998)PGRREEDGDELAR | sp\|P36552\|HEM6_MOUSE | 18.994 | 4 |
| RQIS(1)EDVDGPDNR | sp\|P46935\|NEDD4_MOUSE | 14.755 | 5 |
| KLS(1)PPQKK | sp\|Q8R4S0\|PP14C_MOUSE | 13.113 | 6 |
| DLEFCS(0.805)T(0.195)EEEKETDR | sp\|Q05CL8\|LARP7_MOUSE | 11.396 | 7 |
| TASAVAGKT(1)PDAS(1)PEPK | sp\|Q3TXS7\|PSMD1_MOUSE | 9.777 | 8 |
| LAS(0.002)DDRPS(0.998)PPR | sp\|Q99KG3-2\|RBM10_MOUSE | 9.653 | 9 |
| SES(1)PFTHK | sp\|Q7TPW1\|NEXN_MOUSE | 9.550 | 10 |
| S(0.997)LES(0.003)TNVTDKDGELYCK | sp\|P50462\|CSRP3_MOUSE | 9.457 | 11 |
| RES(1)PPSAER | sp\|Q5SSZ5-2\|TENS3_MOUSE | 8.553 | 12 |
| EPWDGKDGELPVEDDIDLS(1)DVELDDLEKDEL | sp\|Q922R8\|PDIA6_MOUSE | 8.293 | 13 |
| RT(0.929)S(0.071)PVQTR | sp\|P54731\|FAF1_MOUSE | 7.882 | 14 |
| RGT(0.15)GQS(0.813)DDS(0.037)DIWDDTALIK | sp\|P97801\|SMN_MOUSE | 7.860 | 15 |
| VVSS(0.034)T(0.003)S(0.963)EEEEAFTEK | sp\|Q4KMM3-3\|OXR1_MOUSE | 7.603 | 16 |
| KGS(1)GEEEEIDIMELLK | sp\|A2ASS6\|TITIN_MOUSE | 5.144 | 17 |
| RPIS(1)ETR | sp\|A2ASS6\|TITIN_MOUSE | 5.094 | 18 |
| RQIS(1)EDVDGPDNR | sp\|P46935\|NEDD4_MOUSE | 5.045 | 19 |
| ILVPKS(1)PVK | sp\|Q9WTK2-2\|CDYL_MOUSE | 4.977 | 20 |
| DDS(1)PKEYTDLEVSNK | sp\|Q99P72\|RTN4_MOUSE | 4.929 | 21 |
| HSSDINHLVT(0.005)QGRES(0.995)PEGSYTDDANQEVR | sp\|Q8K012-2\|FBP1L_MOUSE | 4.886 | 22 |
| PAEEYEDDAERRS(1)PT(1)PER | sp\|A2ASS6\|TITIN_MOUSE | 4.692 | 23 |
| VHT(0.997)PS(0.003)GAVEECYVSELDSDKHTIR | sp\|Q8VHX6-2\|FLNC_MOUSE | 4.554 | 24 |
| GPPS(1)PPAPVMHS(0.783)PS(0.217)R | sp\|Q9CSN1\|SNW1_MOUSE | 4.518 | 25 |
| KRET(1)DDEGEDD | sp\|Q9EST5\|AN32B_MOUSE | 4.403 | 26 |
| HS(0.055)S(0.888)PT(0.055)EERDEPAY(0.002)PR | sp\|O08648-2\|M3K4_MOUSE | 4.384 | 27 |
| RLS(1)ELLR | sp\|P11499\|HS90B_MOUSE | 4.354 | 28 |
| S(0.005)KDAS(0.993)PINRWS(0.759)PT(0.243)R | sp\|Q61136\|PRP4B_MOUSE | 4.231 | 29 |
| KVEEEQEADEEDVS(1)EEEAEDREGASK | sp\|Q8VBT0\|TMX1_MOUSE | 4.228 | 30 |
| AQTLPTSVVTITSES(0.062)S(0.938)PGKR | sp\|Q62261\|SPTB2_MOUSE | 4.092 | 31 |
| AKS(1)VLKK | sp\|P17809\|GTR1_MOUSE | 4.065 | 32 |
| AISEELDHALNDMT(0.001)S(0.999)I | sp\|P58771\|TPM1_MOUSE | 4.008 | 33 |
| TPFAEDQLHTVPERS(1)PENS(1)PPVK | sp\|Q9QXN0-4\|SHRM3_MOUSE | 4.002 | 34 |
| LSSGPLPQPLGPLT(0.996)PDS(0.004)DIHSGDALPR | sp\|Q3UIZ8-2\|MYLK3_MOUSE | 3.951 | 35 |
| EEQTDT(0.012)S(0.982)DGES(0.006)VTHHIR | sp\|P36916\|GNL1_MOUSE | 3.849 | 36 |
| S(0.863)KS(0.144)ES(0.992)PKEPEQLR | sp\|P49312-2\|ROA1_MOUSE | 3.693 | 37 |
| SLEETLHNVDLS(1)S(1)DDELPRDEEALEDSAEEK | sp\|Q63918\|SDPR_MOUSE | 3.659 | 38 |
| SYS(1)PDGKES(0.994)PS(0.006)DKK | sp\|Q8K310\|MATR3_MOUSE | 3.651 | 39 |
| GHGHS(1)DEEDEEQPR | sp\|Q8N7N5-2\|DCAF8_MOUSE | 3.651 | 40 |
| FKGPGDTSNFDDYEEEEIRVS(1)INEK | sp\|P05132-2\|KAPCA_MOUSE | 3.631 | 41 |
| KGTGDCS(1)DEEVDGKADGADAK | sp\|Q8VDD5\|MYH9_MOUSE | 3.624 | 42 |
| S(0.005)RT(0.17)S(0.825)VQTEDDQLIAGQSAR | sp\|P26231\|CTNA1_MOUSE | 3.491 | 43 |
| AS(0.012)EDES(0.988)DLEDEEEKSQEDTEQKR | sp\|P25206\|MCM3_MOUSE | 3.409 | 44 |
| EAEQGS(1)GEEKEEKEGDLK | sp\|Q6P542\|ABCF1_MOUSE | 3.374 | 45 |
| RPPS(1)PDPNTK | sp\|Q62261\|SPTB2_MOUSE | 3.157 | 46 |
| RLS(1)EQLAHTPTAFK | sp\|Q8BKC8-3\|PI4KB_MOUSE | 3.150 | 47 |
| FAS(1)ENDLPEWK | sp\|P34022\|RANG_MOUSE | 3.142 | 48 |
| AHT(1)PTPGIYMGR | sp\|Q6PFR5\|TRA2A_MOUSE | 3.093 | 49 |
| YLSQLAEEGLKETEGTDS(0.01)PS(0.99)PERGGIGPHLER | sp\|Q3UQS8\|RBM20_MOUSE | 2.957 | 50 |
| RIDFTPVSPAPS(0.983)PT(0.017)R | sp\|Q6NZE7-4\|F122B_MOUSE | 2.923 | 51 |
| KDDSHSAEDS(1)EDEKDDHK | sp\|Q80XU3\|NUCKS_MOUSE | 2.885 | 52 |
| ADPGEDDLGGT(0.002)VDIVES(0.998)EPENEHGVELLDPNNSIR | sp\|P17012\|ZFX_MOUSE | 2.778 | 53 |
| YADQEVPRS(1)PFK | sp\|Q8VHX6-2\|FLNC_MOUSE | 2.748 | 54 |
| RAVVVS(1)PKEENK | sp\|O88874\|CCNK_MOUSE | 2.669 | 55 |
| AS(0.005)S(0.995)PPDRIDIFGR | sp\|Q3UL36-2\|ARGL1_MOUSE | 2.661 | 56 |
| S(0.005)RT(0.17)S(0.825)VQTEDDQLIAGQSAR | sp\|P26231\|CTNA1_MOUSE | 2.624 | 57 |
| RRS(1)PPPR | sp\|P84104-2\|SRSF3_MOUSE | 2.621 | 58 |
| AAAAALS(0.001)GAGAPPAGGGAGGGGS(0.999)PPGGWAVAR | sp\|Q3UCQ1-2\|FOXK2_MOUSE | 2.557 | 59 |
| QESLKS(1)PEEEDQQAFR | sp\|Q6P5H2-2\|NEST_MOUSE | 2.517 | 60 |
| KS(0.006)PS(0.037)GPVKS(0.942)PPLS(0.012)PVGT(0.002)T(0.001)PVK | sp\|Q922M7\|ASHWN_MOUSE | 2.511 | 61 |
| TIPWLENRT(1)PEK | sp\|Q9JI91\|ACTN2_MOUSE | 2.497 | 62 |
| SGPQCS(0.249)S(0.75)PT(0.001)CQEETEDVR | sp\|Q9JHU2\|PALMD_MOUSE | 2.487 | 63 |
| RS(0.035)S(0.965)ANYR | sp\|P48787\|TNNI3_MOUSE | 2.476 | 64 |
| LSGDT(0.192)S(0.807)PPT(0.001)TPSFPR | sp\|Q8BLB7-2\|LMBL3_MOUSE | 2.449 | 65 |
| S(0.006)LS(0.993)ES(0.001)ELIGELSADFDR | sp\|P51125-7\|ICAL_MOUSE | 2.427 | 66 |
| ELALS(1)S(1)PEDLTQDFEELKR | sp\|Q9QYR6\|MAP1A_MOUSE | 2.408 | 67 |
| GSAEGS(1)S(1)DEEGKLVIDEPAKEK | sp\|P51859\|HDGF_MOUSE | 2.399 | 68 |
| ATEEPS(0.002)GT(0.059)GS(0.939)DELIK | sp\|O54724\|PTRF_MOUSE | 2.386 | 69 |
| KAQIITEKT(0.031)S(0.969)PK | sp\|Q99P72\|RTN4_MOUSE | 2.385 | 70 |
| DEILPT(0.028)T(0.966)PIS(0.006)EQK | sp\|P62908\|RS3_MOUSE | 2.351 | 71 |
| AETLSGLGDASAAGAAAVS(0.001)S(0.028)AAS(0.876)ET(0.073)GT(0.022)R | sp\|D3YXK2\|SAFB1_MOUSE | 2.347 | 72 |
| S(1)PS(1)PTPSLPPSWK | sp\|Q8BWB1\|SYP2L_MOUSE | 2.347 | 73 |
| RPYS(1)PEK | sp\|Q8BZR9\|CQ085_MOUSE | 2.332 | 74 |
| FSGEEGEIEDDES(0.802)GT(0.145)ENREEKDS(0.051)LQPS(0.002)AE | sp\|Q569Z6\|TR150_MOUSE | 2.324 | 75 |
| HSTPSNSSNPSGPPS(1)PNSPHR | sp\|Q7TT50\|MRCKB_MOUSE | 2.264 | 76 |
| KAAVLSDS(1)EDDAGNASAK | sp\|Q8C1D8-2\|IWS1_MOUSE | 2.264 | 77 |
| DMPGGFLFDGLS(1)DDEDDFHPSTR | sp\|Q91W67\|UBL7_MOUSE | 2.232 | 78 |
| VVSISSEHLEPIT(0.907)PT(0.093)K | sp\|O55201-2\|SPT5H_MOUSE | 2.202 | 79 |
| ASGQAFELILS(1)PR | sp\|P54227\|STMN1_MOUSE | 2.198 | 80 |
| RLS(0.999)RT(0.001)DLTDYLNR | sp\|Q9CZ13\|QCR1_MOUSE | 2.192 | 81 |
| S(1)VDEVNYWDKQDHPISR | sp\|P50136\|ODBA_MOUSE | 2.175 | 82 |
| VDYSVWDHIEVS(1)DDEDETHPNIDTASLFR | sp\|Q61081\|CDC37_MOUSE | 2.174 | 83 |
| THTPS(0.047)PT(0.188)S(0.764)PKS(0.151)NS(0.848)PQK | sp\|Q9CYT6\|CAP2_MOUSE | 2.167 | 84 |
| LVQAAQMLQS(0.988)DPYS(0.012)VPAR | sp\|Q64727\|VINC_MOUSE | 2.139 | 85 |
| KLEKEEEEGISQES(1)S(1)EEEQ | sp\|P17095-1\|HMGA1_MOUSE | 2.128 | 86 |
| KAS(1)LKDSGEYTCETEASK | sp\|A2AAJ9-2\|OBSCN_MOUSE | 2.125 | 87 |
| LFVIRGS(1)PQQIDHAK | sp\|Q3U0V1\|FUBP2_MOUSE | 2.118 | 88 |
| S(1)GDEMIFDPTMSK | sp\|Q99L45\|IF2B_MOUSE | 2.097 | 89 |
| NTCNSTEKPEELVRT(1)PEEANAGEK | sp\|Q3UZA1-2\|CPZIP_MOUSE | 2.074 | 90 |
| SLSPLSGTT(0.001)DT(0.03)KAES(0.968)PAGR | sp\|Q9JIX8-4\|ACINU_MOUSE | 2.063 | 91 |
| EES(1)PPPAVPEIPKK | sp\|A2ASS6\|TITIN_MOUSE | 2.051 | 92 |
| FIDKDQQPS(1)GS(1)EGEDDDAEAALKK | sp\|Q99J36\|THUM1_MOUSE | 2.041 | 93 |
| HSLS(0.055)GS(0.771)S(0.174)PGMKDTPQTPSR | sp\|Q8BTI8-2\|SRRM2_MOUSE | 2.037 | 94 |
| DVAEEITNYRPTIDTLHEQASALPQAHAES(1)PDVK | sp\|Q62261\|SPTB2_MOUSE | 2.033 | 95 |
| LENEGS(1)DEDIETDVLYSPQMALK | sp\|Q9EQQ9\|NCOAT_MOUSE | 2.016 | 96 |
| AIVSPFHS(0.999)PPSTPSSPGIR | sp\|Q3UZA1-2\|CPZIP_MOUSE | 2.011 | 97 |
| RPLIAS(0.985)PS(0.015)QPPPALR | sp\|Q8VDF2\|UHRF1_MOUSE | 2.010 | 98 |
| NSQEDS(1)EDS(1)EEKDVK | sp\|Q80XU3\|NUCKS_MOUSE | 2.008 | 99 |

# Table S3. List of down-regulated phosphopeptides (fold change < 0.5), as per label-free mass spectrometry in Cypher-knockout mice.

| **Phosphopeptide sequence with phosphosite probability** | **Leading razor protein** | **fold change** | **Rank** |
| --- | --- | --- | --- |
| HALQSASAGS(0.172)GS(0.79)FT(0.038)DVR | sp\|P11276\|FINC_MOUSE | 0.021 | 1 |
| RKES(0.959)T(0.041)PETEEGAPTTSEEK | sp\|Q9DC77\|SMPX_MOUSE | 0.042 | 2 |
| ADES(0.044)S(0.956)DAAGEPQPAPAPVR | sp\|P48787\|TNNI3_MOUSE | 0.056 | 3 |
| APT(0.007)PS(0.014)PVRS(0.979)VS(1)PAGR | sp\|A2ASS6\|TITIN_MOUSE | 0.056 | 4 |
| SPPDQSAVPNT(0.999)PPS(0.597)T(0.404)PVKLEEDLPQEPTSR | sp\|Q9QYC0\|ADDA_MOUSE | 0.076 | 5 |
| VS(0.001)GRT(0.031)S(0.968)PLMLDR | sp\|Q8BTI8-2\|SRRM2_MOUSE | 0.097 | 6 |
| ELS(0.001)HS(0.999)PPRENSFESSLEFK | sp\|Q8BTI8-2\|SRRM2_MOUSE | 0.100 | 7 |
| S(0.005)RS(0.995)PPDRDATEPEPLVDQLIR | sp\|Q8BIA4-4\|FBXW8_MOUSE | 0.114 | 8 |
| KARHDT(1)PDPS(1)PPRR | sp\|Q8R149-2\|BUD13_MOUSE | 0.128 | 9 |
| TQLWAS(0.002)EPGT(0.997)PPVPT(0.001)SLPSQNPILK | sp\|Q99MR6-3\|SRRT_MOUSE | 0.163 | 10 |
| VRHDTPDLS(1)PPRRVR | sp\|Q8R149-2\|BUD13_MOUSE | 0.166 | 11 |
| NRVSSRS(0.965)QT(0.035)EEDCTEELFDFLHAR | sp\|P99028\|QCR6_MOUSE | 0.177 | 12 |
| LEDQMQEDREEGS(0.924)FT(0.076)EGLSEASLPSGLMEGSAEDAEK | sp\|Q63918\|SDPR_MOUSE | 0.178 | 13 |
| HSSLPT(0.033)ES(0.967)DEDIAPAQR | sp\|O54774\|AP3D1_MOUSE | 0.188 | 14 |
| KNS(1)LVLQWKPPVYSGR | sp\|Q62234\|MYOM1_MOUSE | 0.190 | 15 |
| ENS(0.837)AT(0.138)LPPKRS(0.024)PK | sp\|P70670\|NACAM_MOUSE | 0.194 | 16 |
| KGS(1)LNINEFK | sp\|P97352\|S10AD_MOUSE | 0.198 | 17 |
| LPQPS(0.002)S(0.002)GRES(0.996)PRH | sp\|Q8BK30\|NDUV3_MOUSE | 0.199 | 18 |
| RKS(1)HEAEVLK | sp\|P54227\|STMN1_MOUSE | 0.200 | 19 |
| SHETDGGS(1)AHGDEEDDGPHFEPVVPLPDKIEVK | sp\|Q9ERU9\|RBP2_MOUSE | 0.204 | 20 |
| SFSIS(1)PVR | sp\|Q9QX47-4\|SON_MOUSE | 0.207 | 21 |
| S(0.033)GS(0.967)PVKR | sp\|Q93092\|TALDO_MOUSE | 0.207 | 22 |
| VASVS(0.09)RS(0.91)PVDR | sp\|P49452\|CENPC_MOUSE | 0.210 | 23 |
| KLVIIES(1)DLERAEER | sp\|P58771\|TPM1_MOUSE | 0.220 | 24 |
| LS(0.036)S(0.964)VTEDEDQDAALTIVTVLDR | sp\|A2AMM0\|MURC_MOUSE | 0.227 | 25 |
| KVMS(1)PLQSPTK | sp\|Q3UIL6-4\|PKHA7_MOUSE | 0.228 | 26 |
| ILEKFT(1)PK | sp\|A2AAJ9-2\|OBSCN_MOUSE | 0.236 | 27 |
| LKS(1)KES(1)LQEAGK | sp\|P53986\|MOT1_MOUSE | 0.241 | 28 |
| KT(0.062)S(0.938)DPDVILCNSVELIR | sp\|Q7TPE5\|S7A6O_MOUSE | 0.245 | 29 |
| AVT(0.858)S(0.148)PPRVKS(0.993)PEPR | sp\|A2ASS6\|TITIN_MOUSE | 0.246 | 30 |
| KGS(1)DQWTHISTVK | sp\|A2ASS6\|TITIN_MOUSE | 0.249 | 31 |
| S(1)IDDLEDELYAQK | sp\|P58771\|TPM1_MOUSE | 0.251 | 32 |
| AVNS(0.056)T(0.824)RET(0.119)PPKSK | sp\|Q3TEA8-3\|HP1B3_MOUSE | 0.257 | 33 |
| YES(1)IDEDELLASLSPEELKELERELEDIEPDR | sp\|Q3UHZ5\|LMOD2_MOUSE | 0.258 | 34 |
| ELEKPMQS(0.004)KPQS(0.996)PVIQAT(0.151)AGS(0.849)PK | sp\|A2A5R2\|BIG2_MOUSE | 0.261 | 35 |
| GAFS(1)DSEDIDHHSLMAR | sp\|A2ASS6-3\|TITIN_MOUSE | 0.267 | 36 |
| QVDTEEAGMVTAATAS(0.027)NVKAS(0.973)PK | sp\|Q99JF8\|PSIP1_MOUSE | 0.270 | 37 |
| RLS(0.961)LES(0.039)EGANEGATAAPELSALEEAFR | sp\|Q7TQD2\|TPPP_MOUSE | 0.271 | 38 |
| NRPGYVS(1)EEEEDDEDYEMAVK | sp\|Q9ERU9\|RBP2_MOUSE | 0.272 | 39 |
| EEQTDT(0.012)S(0.982)DGES(0.006)VTHHIR | sp\|P36916\|GNL1_MOUSE | 0.275 | 40 |
| LQRPKEES(1)S(1)EDENEVSNILR | sp\|B2RRE7\|OTUD4_MOUSE | 0.281 | 41 |
| AAVGVTGNDIT(0.054)T(0.944)PPNKEPPPS(0.001)PEK | sp\|P27546-3\|MAP4_MOUSE | 0.286 | 42 |
| AELLEEQILQFQS(0.824)RT(0.176)HAMNK | REV__sp\|Q640L5\|CCD18_MOUSE | 0.286 | 43 |
| S(0.135)ET(0.865)APAAPAAPAPVEK | sp\|P43277\|H13_MOUSE | 0.287 | 44 |
| ETPAPSPEGATTAPVQIPPS(1)PR | sp\|P70670\|NACAM_MOUSE | 0.291 | 45 |
| KRLS(1)VELTSSLFR | sp\|Q02248\|CTNB1_MOUSE | 0.293 | 46 |
| PADS(0.046)DS(0.954)DDDPLEAFMAEVEDQAAR | sp\|Q810A7\|DDX42_MOUSE | 0.293 | 47 |
| AT(0.008)S(0.068)PQKS(0.924)PLVPK | sp\|Q9EPC1\|PARVA_MOUSE | 0.294 | 48 |
| PADLDLIQSTPFKPLALKT(1)PPR | sp\|Q6PCP5\|MFF_MOUSE | 0.298 | 49 |
| ANS(1)PPREFPPATAR | sp\|Q8BHL8\|PSMF1_MOUSE | 0.299 | 50 |
| NIS(0.001)LS(0.999)S(1)EEEAEGLAGHPR | sp\|Q5U3K5\|RABL6_MOUSE | 0.299 | 51 |
| GEEDSDVSLAPAVQQMS(0.244)S(0.756)PQPADER | sp\|Q3UQS8\|RBM20_MOUSE | 0.304 | 52 |
| APS(1)PSQPPKK | sp\|Q4VA53\|PDS5B_MOUSE | 0.306 | 53 |
| SRT(0.03)S(0.97)PVTR | sp\|Q8BTI8-2\|SRRM2_MOUSE | 0.312 | 54 |
| FAIQDIS(1)VEETSAK | sp\|Q9JI91\|ACTN2_MOUSE | 0.312 | 55 |
| LRS(0.031)S(0.969)VPGVR | sp\|P20152\|VIME_MOUSE | 0.318 | 56 |
| APLVPHS(0.806)GS(0.194)IEKAEIHEK | sp\|Q8R1N0\|ZN830_MOUSE | 0.322 | 57 |
| RLS(1)LDVEALRR | sp\|E9Q557\|DESP_MOUSE | 0.331 | 58 |
| QKEDVEGVGT(0.212)S(0.788)DGEGAAGLSSDPK | sp\|Q99JP4\|CDC26_MOUSE | 0.334 | 59 |
| KKEPAISSQNS(1)PEAR | sp\|P10711-2\|TCEA1_MOUSE | 0.336 | 60 |
| VGS(1)LDNVGHLPAGGAVK | sp\|P27546-3\|MAP4_MOUSE | 0.336 | 61 |
| AEEKS(1)PVSINVK | sp\|Q52KE7\|CCNL1_MOUSE | 0.338 | 62 |
| ADIDNKEQS(1)ELDQDLEDVEEVEEEETGEETK | sp\|P28656\|NP1L1_MOUSE | 0.367 | 63 |
| RAS(1)PPAAGK | sp\|Q6A0A2-2\|LAR4B_MOUSE | 0.368 | 64 |
| VKESSIIAPVPT(0.712)EDVDT(0.288)PPR | sp\|Q61188-2\|EZH2_MOUSE | 0.372 | 65 |
| EPPT(0.004)IRPNS(0.996)PYDLCNR | sp\|Q5I043-2\|UBP28_MOUSE | 0.374 | 66 |
| S(0.986)PT(0.014)GAQPAAAKPPPLSAK | sp\|E9Q1P8\|I2BP2_MOUSE | 0.379 | 67 |
| NS(0.026)RPS(0.172)S(0.803)PVNTPSSQPPAAK | sp\|Q03173-4\|ENAH_MOUSE | 0.379 | 68 |
| LPAKLS(0.999)VS(0.001)K | sp\|O54724\|PTRF_MOUSE | 0.380 | 69 |
| LGLT(1)PPPEPK | sp\|Q922U1\|PRPF3_MOUSE | 0.387 | 70 |
| ET(0.003)YS(0.01)QLGRVS(0.987)VPEEELDAMLQEGK | sp\|Q9QVP4\|MLRA_MOUSE | 0.389 | 71 |
| GGEDEDGS(1)DEDVVHNEDIHFEPIVSLPEVEVK | sp\|Q9ERU9\|RBP2_MOUSE | 0.390 | 72 |
| QQS(0.001)PS(0.022)PIRHS(0.983)PS(0.994)PVR | sp\|A2ASS6\|TITIN_MOUSE | 0.394 | 73 |
| VKS(1)PEPVTSHPK | sp\|A2ASS6\|TITIN_MOUSE | 0.395 | 74 |
| AELGMNDS(0.959)PS(0.04)QS(0.001)PPVK | sp\|Q9QXG4\|ACSA_MOUSE | 0.396 | 75 |
| KKS(1)HTGEAAAVR | sp\|P59017\|B2L13_MOUSE | 0.396 | 76 |
| EYAENIGDGRS(1)PEFR | sp\|Q9CWL8\|CTBL1_MOUSE | 0.397 | 77 |
| LLRQS(1)PPLAGREEEPGLGDSGIQSTPGSGHAPR | sp\|Q8VDZ4-2\|ZDHC5_MOUSE | 0.403 | 78 |
| PSMS(1)PTPLDR | sp\|Q8BTI8-2\|SRRM2_MOUSE | 0.404 | 79 |
| EADSKPVSQKS(1)PPPAEK | sp\|Q9JLV1\|BAG3_MOUSE | 0.404 | 80 |
| KLS(0.943)S(0.054)AMS(0.002)AAK | sp\|P14152\|MDHC_MOUSE | 0.410 | 81 |
| SHS(1)PPGPSR | sp\|Q3UQS8\|RBM20_MOUSE | 0.413 | 82 |
| GVAKKT(1)PS(1)PIEAER | sp\|A2ASS6\|TITIN_MOUSE | 0.414 | 83 |
| GS(1)LEEQLLQELNNLILR | sp\|Q8R5G7-2\|ARAP3_MOUSE | 0.414 | 84 |
| AAFSKDES(1)KEPIVEVR | sp\|P13595\|NCAM1_MOUSE | 0.414 | 85 |
| AS(1)PGPGGLSGGESLLVK | sp\|P58404-2\|STRN4_MOUSE | 0.418 | 86 |
| HAFSPVAS(0.022)VES(0.918)AS(0.836)GET(0.204)LHS(0.019)PK | sp\|P29699\|FETUA_MOUSE | 0.420 | 87 |
| RGNNS(0.007)AVGS(0.992)NADLTIEEDEEEEPVALQQAQQVR | sp\|Q63918\|SDPR_MOUSE | 0.424 | 88 |
| QQS(1)PSPIR | sp\|A2ASS6\|TITIN_MOUSE | 0.427 | 89 |
| KRVS(1)VELTNSLFK | sp\|Q02257\|PLAK_MOUSE | 0.427 | 90 |
| VESTSVGS(0.019)IS(0.98)PGGAK | sp\|Q9WV92-3\|E41L3_MOUSE | 0.428 | 91 |
| VS(1)PEVGSADVASIAQK | sp\|Q6PGL7\|FAM21_MOUSE | 0.429 | 92 |
| LES(1)PKPVESFASMLR | sp\|Q9CY16\|RT28_MOUSE | 0.434 | 93 |
| PAFVGS(0.001)HDHS(0.987)EES(0.012)QLPR | sp\|Q9QUN9\|DKK3_MOUSE | 0.438 | 94 |
| QQDS(1)QPEEVMDVLEMVESVK | sp\|O35691\|PININ_MOUSE | 0.445 | 95 |
| EVTVGVQPQVRPET(0.018)GQEPS(0.953)PPHS(0.028)DR | sp\|Q9D1T5\|PRR15_MOUSE | 0.447 | 96 |
| KLS(0.999)LS(0.001)EGK | sp\|Q922P9\|GLYR1_MOUSE | 0.447 | 97 |
| S(1)PGHMVILNQTK | sp\|Q8CH25-2\|SLTM_MOUSE | 0.448 | 98 |
| AGSGEDPVLAPS(0.075)GT(0.924)PPPSIPPDETFGGR | sp\|Q9DBD5\|PELP1_MOUSE | 0.451 | 99 |
| KIQPQLPDEDGNHS(1)DKEDEQPQVVVLK | sp\|Q8K039\|K1143_MOUSE | 0.455 | 100 |
| TIS(1)PMVMDAK | sp\|Q64727\|VINC_MOUSE | 0.460 | 101 |
| MELSPS(0.062)RAS(0.938)PGK | sp\|Q9JHU2\|PALMD_MOUSE | 0.460 | 102 |
| KT(0.091)S(0.739)LT(0.142)S(0.028)K | sp\|Q80X50-4\|UBP2L_MOUSE | 0.469 | 103 |
| SLYS(0.005)S(0.174)S(0.821)PGGAYVTR | sp\|P20152\|VIME_MOUSE | 0.469 | 104 |
| RAS(1)GQAFELILSPR | sp\|P54227\|STMN1_MOUSE | 0.476 | 105 |
| ESVPDFPLS(1)PPK | sp\|P54227\|STMN1_MOUSE | 0.477 | 106 |
| KT(0.268)S(0.728)GPPVS(0.003)ELIT(0.001)K | sp\|P43274\|H14_MOUSE | 0.479 | 107 |
| T(1)PPPTT(0.004)PKT(0.996)PPPVAPKPGSR | sp\|Q8BWB1\|SYP2L_MOUSE | 0.481 | 108 |
| VHAYFAPVT(1)PPPSVGGSR | sp\|Q9CZV8-4\|FXL20_MOUSE | 0.482 | 109 |
| AIDS(0.008)S(0.986)ET(0.006)EDFDSEKEISQIFSK | sp\|Q9ET54\|PALLD_MOUSE | 0.485 | 110 |
| HSLS(1)LDDIR | sp\|Q8C0V9-2\|FRMD6_MOUSE | 0.487 | 111 |
| VEGTEIVKPS(1)PK | sp\|P97868-2\|RBBP6_MOUSE | 0.489 | 112 |
| RT(0.017)S(0.983)MGGTQQQFVEGVR | sp\|Q02248\|CTNB1_MOUSE | 0.489 | 113 |
| S(0.027)RT(0.973)PPSAPSQSR | sp\|Q8BTI8-2\|SRRM2_MOUSE | 0.494 | 114 |
| S(1)PGGPGPLTLK | sp\|Q62523\|ZYX_MOUSE | 0.498 | 115 |
| RS(0.035)S(0.965)ANYR | sp\|P48787\|TNNI3_MOUSE | 0.498 | 116 |
| VDNLTYRT(0.201)S(0.799)PDTLR | sp\|Q62093\|SRSF2_MOUSE | 0.499 | 117 |
